# Associations between diabetes-related genetic risk scores and residual beta cell function in type 1 diabetes: the GUTDM1 study

**DOI:** 10.1007/s00125-024-06204-6

**Published:** 2024-06-26

**Authors:** Coco M. Fuhri Snethlage, Manon Balvers, Bart Ferwerda, Elena Rampanelli, Pleun de Groen, Bart O. Roep, Hilde Herrema, Timothy J. McDonald, Daniël H. van Raalte, Michael N. Weedon, Richard A. Oram, Max Nieuwdorp, Nordin M. J. Hanssen

**Affiliations:** 1 Department of (Experimental) Vascular and Internal Medicine, Amsterdam UMC, University of Amsterdam, Amsterdam, the Netherlands; 2 Department of Clinical Epidemiology and Biostatistics, Amsterdam UMC, Amsterdam, the Netherlands; 3 Leids Universitair Medisch Centrum, Internal Medicine, Leiden, the Netherlands; 4 Peninsula College of Medicine and Dentistry, Peninsula NIHR Clinical Research Facility, Exeter, Devon UK; 5 Department of Endocrinology and Metabolism, Amsterdam UMC, Amsterdam, the Netherlands; 6 Diabeter Center Amsterdam, Amsterdam, the Netherlands

**Keywords:** CGM, Polygenic risk score, Residual beta cell function, Type 1 diabetes

## Abstract

**Aims/hypothesis:**

Use of genetic risk scores (GRS) may help to distinguish between type 1 diabetes and type 2 diabetes, but less is known about whether GRS are associated with disease severity or progression after diagnosis. Therefore, we tested whether GRS are associated with residual beta cell function and glycaemic control in individuals with type 1 diabetes.

**Methods:**

Immunochip arrays and TOPMed were used to genotype a cross-sectional cohort (*n*=479, age 41.7 ± 14.9 years, duration of diabetes 16.0 years [IQR 6.0–29.0], HbA_1c_ 55.6 ± 12.2 mmol/mol). Several GRS, which were originally developed to assess genetic risk of type 1 diabetes (GRS-1, GRS-2) and type 2 diabetes (GRS-T2D), were calculated. GRS-C1 and GRS-C2 were based on SNPs that have previously been shown to be associated with residual beta cell function. Regression models were used to investigate the association between GRS and residual beta cell function, assessed using the urinary C-peptide/creatinine ratio, and the association between GRS and continuous glucose monitor metrics.

**Results:**

Higher GRS-1 and higher GRS-2 both showed a significant association with undetectable UCPCR (OR 0.78; 95% CI 0.69, 0.89 and OR 0.84: 95% CI 0.75, 0.93, respectively), which were attenuated after correction for sex and age of onset (GRS-2) and disease duration (GRS-1). Higher GRS-C2 was associated with detectable urinary C-peptide/creatinine ratio (≥0.01 nmol/mmol) after correction for sex and age of onset (OR 6.95; 95% CI 1.19, 40.75). A higher GRS-T2D was associated with less time below range (TBR) (OR for TBR<4% 1.41; 95% CI 1.01 to 1.96) and lower glucose coefficient of variance (β −1.53; 95% CI −2.76, −0.29).

**Conclusions/interpretation:**

Diabetes-related GRS are associated with residual beta cell function in individuals with type 1 diabetes. These findings suggest some genetic contribution to preservation of beta cell function.

**Graphical Abstract:**

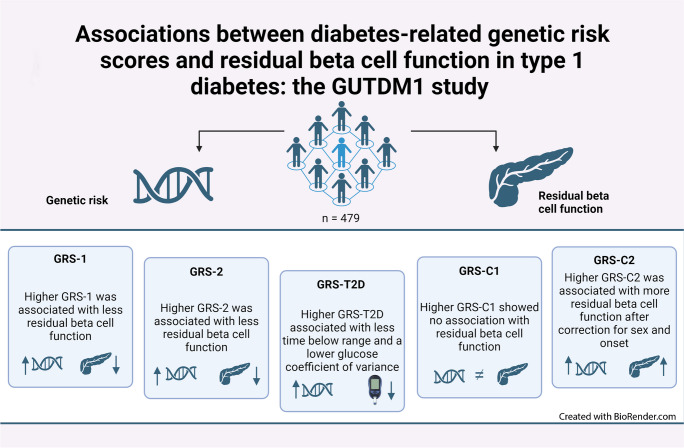

**Supplementary Information:**

The online version of this article (10.1007/s00125-024-06204-6) contains peer-reviewed but unedited supplementary material.


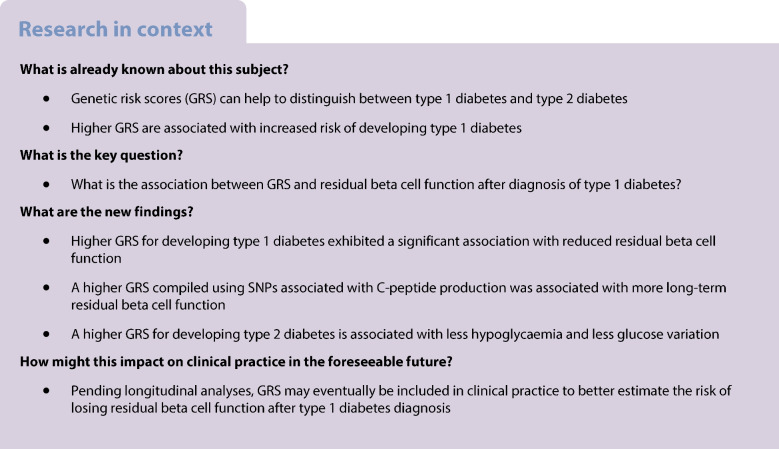


## Introduction

Type 1 diabetes is a T cell-mediated auto-immune disease that results in the destruction of pancreatic beta cells and lifelong insulin dependency [1]. The exact underlying aetiology of type 1 diabetes is still unknown, but it is generally considered to involve a complex interplay between genetic and environmental factors [2–5]. Even with tight glycaemic control, type 1 diabetes has a high morbidity and mortality rate, with a five times higher risk of CVD and a mean reduced life expectancy of 12 years [6–9].

Recently, it has become apparent that a large proportion of individuals with type 1 diabetes have residual beta cell function [10, 11], and that this is associated with fewer hypoglycaemic events and long-term complications, and better daily glycaemic control [12–14]. New therapeutic strategies such as treatment with verapamil, teplizumab, pleconaril or ribavirin or faecal transplantations have attempted to preserve residual beta cell function [15–17]. It is vital to understand which individuals maintain residual beta cell function and whether genetic or environmental factors are implicated.

While the genetic susceptibility to type 1 diabetes is high [18], with more than 136 risk loci identified [19], less is known about the influence of genetic factors on residual beta cell function in longstanding type 1 diabetes. Previous studies have shown that residual beta cell function is influenced by SNPs in the HLA region that are distinct from those determining age of onset [20, 21]. Additionally, a handful of other loci have been associated with residual beta cell function [20–22].

Genetic risk scores (GRS) are used to evaluate someone’s risk for a certain phenotype/disease based on the presence of associated variants/loci in an individual. For type 1 diabetes, multiple GRS have been developed that can distinguish between diabetes types and may be used to screen for individuals at risk for type 1 diabetes [23–25]. Potentially, diabetes-related GRS could also assist in predicting the maintenance of residual beta cell function in type 1 diabetes patients, with applications for either trial design or prediction of treatment response.

Given these considerations, we aimed to investigate the association between several existing diabetes-related GRS and residual beta cell function in our own heterogeneous type 1 diabetes cohort. We also investigated the association between residual beta cell function and a new genetic risk score based on SNPs previously related to beta cell function. Lastly, we assessed the various GRS for associations with continuous glucose monitor (CGM) metrics.

## Methods

### Participant recruitment

Five hundred individuals participated in the GUTDM1 cohort, with data collected from November 2020 to October 2022. The GUTDM1 cohort is a cross-sectional study in the Netherlands designed to investigate the interplay between genetic and environmental factors in the maintenance of residual beta cell function in type 1 diabetes [12]. Participant recruitment and informed consent procedures adhered to the Declaration of Helsinki, and received approval from the local Medical Ethics Committee of the Amsterdam University Medical Centre. Participants were eligible for inclusion when they were above 18 years and willing/able to sign informed consent. Individuals were excluded if they had active infection or a total colectomy. Overall, the study was representative for the general Dutch population, however the study included more women than men and social economic status was unknown. All participants had a type 1 diabetes diagnosis by their own physician prior to the study visit in accordance with EASD/ADA guidelines [26, 27]. The gender of participants was self-reported and in concordance with the sex based on the genetic data of the single nucleotide polymorphism Immunochip array.

### Data collection and study visit

Study visits were performed at the Academic Medical Centre of the Amsterdam University Medical Centre. We measured stimulated urinary C-peptide/creatinine ratio (UCPCR), fasting glucagon and calculated CGM metrics as described in the electronic supplementary material (ESM) Methods [28–30].

### Generation of GRS

Methods for DNA isolation, genotyping and quality control, determining genetic ancestry and imputation are described in ESM Methods [12, 31–33]. We calculated five GRS based on known associations with incident type 1 diabetes (GRS-1 and GRS-2), type 2 diabetes (GRS-T2D) and C-peptide secretion levels (GRS-C1 and GRS-C2).

#### GRS-1

This GRS was originally designed to distinguish between type 1 diabetes and type 2 diabetes in young adults [34]. It comprises 30 SNPs, including both HLA and non-HLA SNPs (ESM Table 1). Two SNPs were used to assess the high-risk *DR3* and *DR4-DQ8* haplotype and incorporated as one term in the model (ESM Table 2). We used rs9273369 as a proxy for rs2187668, which was not present in our data after imputation. Final GRS-1 was calculated as shown in Eq. 1:

1 $$\text{GRS}-1=\text{HLA risk genotype}+ {\sum }_{i=1}^{28}{\beta }_{i}\times {s}_{i}$$

in which *β*_*i*_ represents the weight of the non-*DR3* and non-*DR4-DQ8* SNP_*i*_, and *s*_*i*_ is the number of effect alleles (0, 1 or 2) for this SNP. All SNPs had high imputation quality (all *R*^2^>0.95) and were present in all samples.

#### GRS-2

This GRS is an improved version of GRS-1 to predict type 1 diabetes in newborn screening studies and to better discriminate between diabetes subtypes [23]. Compared with GRS-1, HLA alleles and their interactions were more completely incorporated, and GRS-2 contained newly discovered non-HLA loci. The GRS-2 consists of 67 SNPs (ESM Table 3). Fourteen SNPs were used to assess *DR-DQ* haplotypes, which resulted in one overall DR-DQ score for GRS-2. *DR-DQ* haplotypes were determined per person. If more than two candidate haplotypes could be assigned, the two most likely haplotypes were selected based on allele frequencies in the general population (ESM Table 4). If two haplotypes were known to have an interaction (i.e. have a different effect when present together compared with alone), the DR-DQ score was derived from ESM Table 5, otherwise the DR-DQ score was calculated using Eq. 2:

2 $$DR-DQ\, score={\sum }_{k=0}^{a}\,{\beta }_{haplotype}\times {n}_{i}$$

In Eq. 2, *β*_*haplotype*_ is the weight for the identified *DR-DQ* haplotype from ESM Table 6, while *n*_*i*_ is the number of risk alleles present for this haplotype. The final GRS-2 is calculated by adding the DR-DQ score to the individual scores from the other HLA SNPs (21 SNPs) and non-HLA SNPs (32 SNPs) as shown in Eq. 3:

3 $$\text{GRS}-2=DR-DQ\, score+ {\sum }_{i=1}^{53}\,{\beta }_{i}\times {s}_{i}$$

In Eq. 3, *β*_*i*_ represents the weight (log OR) of the non-*DR-DQ* SNP_*i*_, and *s*_*i*_ is the number of effect alleles (0, 1 or 2) for this SNP, weight values are presented in ESM Table 3. The lowest imputation quality across all SNPs was 0.81. SNP rs17840116 was missing in our dataset and therefore not taken into account. All other SNPs were present in all samples.

#### GRS-T2D

To investigate whether type 1 diabetes participants with a higher GRS for type 2 diabetes have a different course of disease, we included a GRS for type 2 diabetes (GRS-T2D). GRS-T2D is based on the 403 distinct type 2 diabetes association signals identified in a genome-wide association study (GWAS) performed in people of white European ancestry [24]. We used the summary statistics from all SNPs that were present in our dataset after imputation, which resulted in the inclusion of 237 variants (ESM Table 7). The final GRS-T2D was obtained using Eq. 4:

4 $$\text{GRS}-\text{T}2\text{D}={\sum }_{i=1}^{237}{\beta }_{i}\times {s}_{i}$$

In Eq. 4, *β*_*i*_ represents the weight (log OR) of SNP_*i*_, and *s*_*i*_ represents the number of effect alleles (0, 1 or 2) for this SNP, present in ESM Table 7. The lowest imputation quality across all SNPs was 0.7. All variants were present in all samples.

#### GRS-C1 and GRS-C2

Finally, we generated two GRS based on the targeted GWAS performed by Harsunen et al [20]. These investigators tested known SNPs associated with type 1 diabetes (123 SNPs), type 2 diabetes (363 SNPs) and C-peptide (six SNPs) for association with random non-fasting serum C-peptide levels in a Finnish cohort of type 1 diabetes patients. We used the summary statistics of all SNPs with *p*≤0.05 that were not in linkage disequilibrium (based on the SNPclip Tool in the European 1000G population, with thresholds minor allele frequency [MAF]=0.01 and *R*^2^=0.1 [35]) and present after imputation. This resulted in GRS-C1, consisting of 21 SNPs. In addition, we created a GRS based on a stricter *p* value cut-off (*p*<0.005), called GRS-C2, which contained seven SNPs (ESM Table 8). Both GRS were obtained in the same way as Eq. 4, but replacing the type 2 diabetes SNPs with the C-peptide SNPs. The lowest imputation quality across all SNPs was 0.71. All variants were present in all samples.

### Statistics

Clinical and anthropometric values are summarised as mean ± SD or as median (IQR) for normally and non-normally distributed values, respectively. Categorical variables are presented as percentages. Correlations between GRS and continuous outcomes were assessed using Spearman correlations.

Residual beta cell function was assessed using a binary outcome variable (i.e. undetectable vs detectable UCPCR; <0.01 nmol/mmol vs ≥0.01 nmol/mmol) in logistic regression analysis, because UCPCR itself showed a very skewed distribution (ESM Fig. 1). Univariate analyses were performed for all GRS. In addition, models were additively adjusted for sex (model 2), sex and age of onset (model 3; i.e. model 2 + age of onset) and sex, age of onset and duration of disease (model 4; i.e. model 3 + duration). A directed acyclic graph was designed to show potential biasing paths (ESM Fig. 2). Similar regression models were performed for time in range (TIR≥70%), time above range (TAR<25%) and time below range (TBR<4%) as binary outcomes [30], and glucose coefficient of variance (GCV) and HbA_1c_ as continuous outcomes.

Sensitivity analyses were performed in all participants with determined European genetic ancestry (ESM Methods/Determining genetic ancestry) and all participants with disease duration longer than 7 years. Covariates related to type 1 diabetes were tested for significant differences across tertiles for GRS-1 using ANOVA or the Kruskal–Wallis test (normally and non-normally distributed continuous covariates, respectively) or the χ^2^ test (categorical covariates).

All statistical analyses were performed in R 3.6.2 (using RStudio version 1.2.5033); a two-sided *p* value of <0.05 was considered statistically significant.

## Results

Of the 500 individuals participating in the GUTDM1 cohort, 479 were eligible for inclusion in the current analysis (ESM Fig. 3). The mean age (± SD) was 41.7 ± 14.9 years, the median BMI was 24.5 kg/m^2^ (IQR 22.6–27.4), and the median duration of type 1 diabetes was 16.0 years (IQR 6.0–29.0). Participants had an HbA_1c_ of 55.6 ± 12.2 mmol/mol (7.2 ± 3.3%; means ± SD) and a median TIR of 66.0% (IQR 51.0–80.0). Across all participants, 50.1% had a detectable UCPCR (≥0.01 nmol/mmol). Participants with detectable UCPCR had a lower BMI and were younger, with fewer complications and better glycaemic control (Table 1).

**Table 1:** Participant characteristics

Characteristics	Undetectable UCPCR^a^	Detectable UCPCR^a^	*p* value
*N*	239	240	
Male (%)	38	36	0.750
Age, years	44.0 (29.5–54.0)	37.5 (27.0–52.0)	0.019
BMI, kg/m^2^	25.0 (23.2–28.2)	24.1 (21.9–26.5)	<0.001
Onset of type 1 diabetes, years	14.0 (8.0–22.0)	28.0 (18.0–41.0)	<0.001
Duration of type 1 diabetes, years	25.0 (17.0–35.5)	7.0 (3.0–14.0)	<0.001
TIR, %	60.0 (48.0–75.0)	72.0 (56.0–87.0)	<0.001
TAR, %	35.5 (22.0–49.0)	24.0 (10.0–41.5)	<0.001
TBR, %	2.0 (1.0–5.0)	1.0 (0.8–4.0)	0.001
GCV, %	36.0 ± 7.8	32.2 ± 7.8	<0.001
Fasting C-peptide, nmol/l	0.05 (0.05–0.05)	0.08 (0.05–0.16)	<0.001
HbA_1c_, mmol/mol	56.8 ± 11.3	54.3 ± 13.0	0.027
HbA_1c_, %	7.3 ± 3.2	7.1 ± 3.3	0.027
Fasting glucose, mmol/l	8.7 (7.2–11.3)	7.8 (6.2–10.5)	0.008
UCPCR, nmol/mmol	0.00 (0.00–0.00)	0.41 (0.10–1.03)	<0.001
Fasting glucagon, ng/l	122 (76–189)	160 (97–270)	<0.001
GGR	0.31 (0.22–0.53)	0.48 (0.28–0.78)	<0.001
Sensor type			
Freestyle Libre 2	67	82	<0.001
Dexcom G6	20	7	
Medtronic Guardian	12	11	
Insulin pump			<0.001
No	39	61	
Manual	34	29	
Predictive low-glucose suspend	2	0	
Hybrid closed loop	20	8	
DIY closed loop	5	3	
Total insulin, U/day	42.6 (30.9–56.0)	32.0 (21.0–46.8)	<0.001
Smoking	10	11	0.775
Alcohol, units/day	0.29 (0.00–0.86)	0.29 (0.00–0.86)	0.426
Any co-medication	46	41	0.292
Albumin-to-creatinine ratio	0.44 (0.00–0.93)	0.32 (0.00–0.74)	0.005
Retinopathy	51	15	<0.001
CVD	23	13	0.005
Systolic BP, mmHg	131.3 ± 16.6	127.8 ± 17.2	0.027
eGFR^b^, ml/min per 1.73 m^2^	90.0 (88.5–90.0)	90.0 (89.0–90.0)	0.983

Values are mean ± SD or median (IQR) for continuous variables, or % for categorical variables

^a^Values for UCPCR are defined as undetectable (<0.01 nmol/mmol) or detectable (values ≥0.01 nmol/mmol)

^b^Calculated using the CKD-EPI 2021 method

GGR, glucagon to glucose ratio

The clinical characteristics of participants were divided into tertiles of genetic risk of type 1 diabetes (GRS-1) and assessed for potential previously unknown confounders, such as age of onset and duration of diabetes. The results are shown in ESM Table 9.

### Associations between various GRS and odds of having a detectable beta cell function

We correlated type 1 diabetes-related GRS (GRS-1 and GRS-2), type 2 diabetes-related GRS (GRS-T2D) and newly created GRS based on SNPs associated with residual beta cell function (GRS-C1 and GRS-C2) with UCPCR and age of onset of type 1 diabetes (Table 2). We found that a higher genetic risk for type 1 diabetes (indicated by both GRS-1 and GRS-2), a lower genetic risk for type 2 diabetes (indicated by GRS-T2D) and earlier age of onset significantly correlated with a lower UCPCR. Furthermore, both higher GRS-1 and higher GRS-2 were significantly correlated with lower GRS-C2 (Table 2).

**Table 2:** Correlation matrix between the calculated GRS, TIR, TBR, TAR, GCV, HbA_1c_, onset of disease and UCPCR on continuous scales assessed using complete-case Spearman correlations

	UCPCR	TIR	TBR	TAR	GCV	HbA_1c_	GRS−1	GRS−2	GRS-T2D	GRS-C1	GRS-C2	Onset
UCPCR	1											
TIR	0.331***	1										
TBR	−0.225***	−0.010	1									
TAR	−0.305***	−0.980***	−0.139**	1								
GCV	−0.353***	−0.535***	0.574***	0.441***	1							
HbA_1c_	−0.193***	−0.694***	−0.149**	0.714***	0.347***	1						
GRS−1	−0.128**	−0.044	0.039	0.044	0.047	0.036	1					
GRS−2	−0.125**	−0.065	0.074	0.054	0.106*	−0.003	0.762***	1				
GRS-T2D	0.097*	0.045	−0.077	−0.041	−0.138**	−0.004	−0.035	−0.015	1			
GRS-C1	0.084	0.035	−0.025	−0.029	−0.005	0.011	−0.079	−0.084	0.055	1		
GRS-C2	0.079	0.035	−0.027	−0.022	−0.047	−0.021	−0.093*	−0.144**	−0.010	0.692***	1	
Onset	0.508***	0.208***	−0.125**	−0.202***	−0.259***	−0.067	−0.122**	−0.173***	0.052	0.029	−0.004	1

Standard statistical notation was used: *p<0.05, **p<0.01, ***p<0.001

**Fig. 1:**
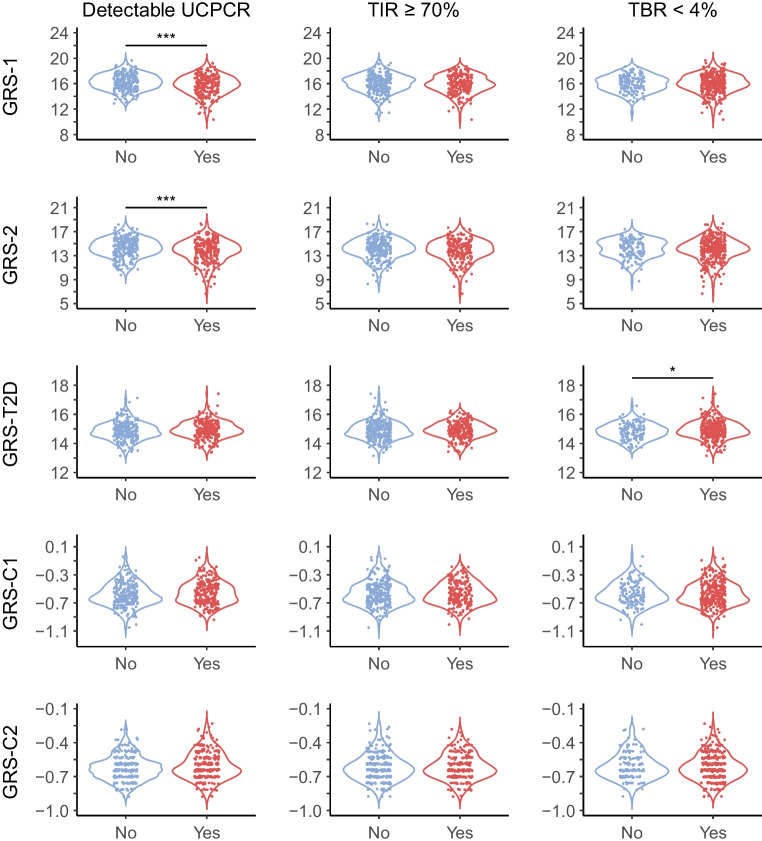
Violin plot visualisation of all GRS by binary UCPCR status, binary TIR status and TBR status. If participants had a detectable UCPCR, they were classified as yes, otherwise classified as no. TIR ≥70% or TBR <4% were classified as yes, otherwise no. Significant differences between groups were tested using unpaired Student’s *t* test and are indicated as ***p<0.001 or *p<0.05

We subsequently assessed the relationship between the various GRS and detectable UCPCR (Table 3 and Fig. 1). Individuals with a higher genetic risk for type 1 diabetes (indicated by higher GRS-1 or higher GRS-2) had lower odds of detectable UCPCR in unadjusted models (model 1). While adjustment for sex (model 2) did not attenuate the associations, additional correction for age of onset (model 3) did, and removed statistical significance for GRS-2. Genetic risk for type 2 diabetes, indicated by GRS-T2D, was not significantly associated with detectable UCPCR in any of the models (Table 3 and Fig. 1). The association between the newly created GRS-C2 and UCPCR was not significant in the unadjusted model (Table 3 and Fig. 1) but became significant after correction for sex and age of onset (model 3, Table 3), showing that participants with a higher GRS for C-peptide secretion were more likely to have a detectable UCPCR. However, GRS-C1, the other GRS related to C-peptide secretion, was not significantly associated with detectable UCPCR. For all GRS, adjustment for duration of type 1 diabetes in the fully adjusted model (model 4) had a minor impact on the point estimates, but overall attenuated associations to the extent that they were no longer statistically significant.

**Table 3:** Associations between GRS and UCPCR (nmol/mmol), TIR, TAR, TBR, GCV and HbA_1c_

	UCPCR (detectable)	TIR ≥70%	TBR <4%	TAR <25%	GCV	HbA_1c_
Model 1: unadjusted						
GRS-1	0.78 (0.69, 0.89)***	0.90 (0.80, 1.03)	0.98 (0.85, 1.12)	0.92 (0.81, 1.04)	0.24 (−0.29, 0.78)	−0.02 (−0.79, 0.75)
GRS-2	0.84 (0.75, 0.93)***	0.91 (0.82, 1.01)	0.94 (0.84, 1.06)	0.92 (0.83, 1.02)	0.47 (0.04, 0.90)*	−0.09 (−0.72, 0.53)
GRS-T2D	1.26 (0.94, 1.70)	1.01 (0.75, 1.36)	1.41 (1.01, 1.96)*	1.11 (0.82, 1.50)	−1.53 (−2.76, −0.29)*	−0.24 (−2.06, 1.59)
GRS-C1	2.78 (0.94, 8.16)	1.01 (0.35, 2.97)	1.24 (0.38, 4.00)	0.58 (0.20, 1.74)	−0.94 (−5.39, 3.51)	0.22 (−6.33, 6.78)
GRS-C2	4.62 (0.96, 22.15)	1.87 (0.39, 8.90)	0.67 (0.12, 3.62)	0.53 (0.11, 2.59)	−2.03 (−8.48, 4.42)	−2.37 (−11.87, 7.13)
Model 2: adjusted for sex						
GRS-1	0.78 (0.69, 0.89)***	0.90 (0.80, 1.03)	0.98 (0.85, 1.12)	0.92 (0.81, 1.04)	0.23 (−0.30, 0.76)	−0.01 (−0.78, 0.76)
GRS-2	0.84 (0.75, 0.93)***	0.91 (0.82, 1.01)	0.94 (0.84, 1.06)	0.92 (0.83, 1.02)	0.47 (0.04, 0.90)*	−0.10 (−0.72, 0.53)
GRS-T2D	1.26 (0.93, 1.70)	1.01 (0.75, 1.35)	1.40 (1.01, 1.96)*	1.11 (0.82, 1.50)	−1.51 (−2.74, −0.27)*	−0.25 (−2.07, 1.57)
GRS-C1	2.77 (0.94, 8.16)	1.01 (0.34, 2.96)	1.23 (0.38, 4.01)	0.58 (0.20, 1.74)	−0.93 (−5.38, 3.52)	0.19 (−6.36, 6.73)
GRS-C2	4.66 (0.97, 22.35)	1.88 (0.39, 8.96)	0.68 (0.13, 3.72)	0.53 (0.11, 2.58)	−2.10 (−8.55, 4.35)	−2.21 (−11.69, 7.28)
Model 3: adjusted for sex and age at onset						
GRS-1	0.82 (0.70, 0.94)**	0.92 (0.81, 1.05)	0.99 (0.86, 1.14)	0.94 (0.82, 1.07)	0.01 (−0.51, 0.53)	−0.04 (−0.82, 0.74)
GRS-2	0.90 (0.80, 1.01)	0.93 (0.84, 1.04)	0.96 (0.86, 1.08)	0.94 (0.85, 1.05)	0.24 (−0.19, 0.66)	−0.14 (−0.77, 0.50)
GRS-T2D	1.22 (0.87, 1.71)	0.98 (0.73, 1.33)	1.39 (0.99, 1.94)	1.09 (0.80, 1.47)	−1.35 (−2.54, −0.16)*	−0.23 (−2.05, 1.60)
GRS-C1	3.05 (0.91, 10.25)	0.96 (0.32, 2.84)	1.18 (0.36, 3.87)	0.54 (0.18, 1.64)	−0.61 (−4.90, 3.67)	0.27 (−6.29, 6.82)
GRS-C2	6.95 (1.19, 40.75)*	1.85 (0.38, 8.96)	0.67 (0.12, 3.67)	0.51 (0.10, 2.53)	−1.68 (−7.89, 4.53)	−2.16 (−11.66, 7.33)
Model 4: adjusted for sex, age at onset and disease duration						
GRS-1	0.86 (0.73, 1.01)	0.93 (0.82, 1.06)	1.00 (0.87, 1.15)	0.95 (0.83, 1.09)	−0.04 (−0.56, 0.48)	−0.11 (−0.89, 0.67)
GRS-2	0.93 (0.82, 1.06)	0.94 (0.84, 1.04)	0.96 (0.86, 1.08)	0.95 (0.86, 1.06)	0.20 (−0.22, 0.62)	−0.18 (−0.82, 0.45)
GRS-T2D	1.15 (0.79, 1.65)	0.97 (0.72, 1.32)	1.38 (0.99, 1.93)	1.07 (0.79, 1.45)	−1.29 (−2.48, −0.10)*	−0.15 (−1.97, 1.67)
GRS-C1	1.60 (0.40, 6.31)	0.87 (0.29, 2.61)	1.13 (0.34, 3.71)	0.47 (0.15, 1.43)	−0.05 (−4.36, 4.26)	0.94 (−5.64, 7.52)
GRS-C2	2.54 (0.35–18.39)	1.63 (0.33–7.97)	0.61 (0.11–3.40)	0.41 (0.08, 2.05)	−0.80 (−7.07, 5.47)	−1.15 (−10.70, 8.40)

UCPCR was categorised as detectable/not detectable, TIR as above or below 70%, TAR as above or below 25%, TBR as above or below 4%. HbA_1c_ and GCV were modelled as continuous outcomes. This table depicts logistic or linear regression models where the OR (for logistic regression) or β (for linear regression) are expressed per score point of the GRS. Values are OR (95% CI) for UCPCR, TIR, TBR and TAR, and β (95% CI) for GCV and HbA_1c_

Standard statistical notation was used: *p<0.05, **p<0.01, ***p<0.001

Next, we performed a principal component analysis to determine genetic ancestry (ESM Fig. 4), and thereafter performed a sensitivity analysis by excluding individuals (*n*=20) classified as having non-European genetic ancestry, as some GRS have not been validated in this group. The associations remained overall similar for GRS-1 and GRS-2 in all models (ESM Fig. 5 and ESM Table 10). The association between higher GRS-C2 and detectable UCPCR was significant in the unadjusted model and remained significant in model 2 and 3, but not in model 4. To account for the rapid decrease in UCPCR in the first 7 years after diagnosis [36], we performed another sensitivity analysis including only individuals with a type 1 diabetes duration longer than 7 years. In this analysis, only a higher GRS-1 was significantly associated with undetectable UCPCR in models 1, 2 and 3 (ESM Fig. 6 and ESM Table 11).

### Performance of various GRS on CGM metrics

To assess the relationship between glycaemic control and GRS, we investigated the correlation between the various GRS and TIR, TBR and TAR (all *n*=475), GCV (*n*=437) and HbA_1c_ (*n*=477) (ESM Fig. 3). We found no significant correlation between any of the GRS and the continuous values for either TIR, TAR, TBR or HbA_1c_ in Spearman correlations (Table 2). However, a higher GCV was significantly correlated with a higher risk of type 1 diabetes (i.e. higher GRS-2) and lower risk of type 2 diabetes (i.e. lower GRS-T2D).

In agreement with the correlation analysis, TIR, TAR and HbA_1c_ were not significantly associated with any of the GRS in our regression models (Table 3 and Fig. 1). This was confirmed in our sensitivity analyses for only participants with European genetic ancestry and participants with a type 1 diabetes duration of more than 7 years (ESM Tables 10 and 11).

While the Spearman correlation between less TBR and higher GRS-T2D was not significant, a higher GRS-T2D was significantly associated with less TBR in the unadjusted logistic regression model (model 1), even after correction for sex (model 2), but not age of onset and duration (models 3 and 4, Table 3). The results were overall similar for participants with European genetic ancestry, but were not significant (ESM Table 10). However, when examining participants with a disease duration of more than 7 years, we found that a higher GRS-T2D was significantly associated with less TBR in models 2, 3 and 4 (ESM Table 11).

Higher GRS-2 showed a significant association with higher GCV in the linear regression models. This association remained significant after correction for sex, but not after correction for age of onset nor type 1 diabetes duration (Table 3). In contrast, a higher GRS-T2D was associated with lower GCV, and this association remained significant in all models. The subset analysis of participants who had a type 1 diabetes duration of more than 7 years, identified even stronger associations, which were also all significant (ESM Table 11).

After including an interaction term between sex and GRS (model 5), we observed a significant difference in the effect of GRS on TAR (GRS-1 and GRS-2), GCV (GRS-1) and HbA_1c_ (GRS-1 and GRS-C2) between men and women. Generally, in women, a higher GRS-1 indicated poorer glycaemic control (higher TAR, higher HbA_1c_ and higher GCV), while the direction was opposite in men. Similar phenomena were observed for GRS-2 and GRS-C2 (data not shown).

## Discussion

In this study, we found that residual beta cell function was associated with several diabetes-related GRS. In particular, a high genetic risk for type 1 diabetes (indicated by high GRS-1 or high GRS-2) and a low genetic risk for residual C-peptide secretion (indicated by low GRS-C2) were associated with undetectable residual beta cell function (UCPCR <0.01 nmol/mmol). In addition, a higher genetic risk for type 2 diabetes was associated with better glycaemic control, indicated by less TBR and lower variance in glucose levels.

GRS for type 1 diabetes are increasingly being used to differentiate between types of diabetes [25, 34], and have been proposed as screening tools for type 1 diabetes [23]. In our study, high GRS-1 or high GRS-2 were significantly associated with undetectable C-peptide in the unadjusted models, but this was attenuated after additive correction for sex, age of onset (GRS-2) and duration (GRS-1). A previous study found that a higher GRS-1 was associated with lower odds of detecting random C-peptide levels after correcting for age of onset and duration, although this association was not significant [37]. Another study did find a significant association between a higher type 1 diabetes GRS score (consisting of non-HLA SNPs) and lower stimulated C-peptide, but no significant association for GRS-1 [22, 38]. Two other cohort studies created their own type 1 diabetes GRS and showed that individuals with a lower type 1 diabetes GRS had significantly higher random C-peptide levels [20, 21], supporting our findings. This association was largely dependent on risk SNPs in the HLA region, as the association disappeared when the HLA region was excluded. However, the non-HLA genetic risk score did show differences between subsets of participants with the highest and lowest random C-peptide levels without adjusting for confounders [20].

GRS-2 mainly differs from GRS-1 in the way the HLA risk (especially the *DR-DQ* haplotype) is captured [23]. As HLA serotype risk is strongly associated with a lower age of onset and development of type 1 diabetes, this may explain the observed differences across the scores in our cohort. Combining our results with those of the above-listed studies, it appears that residual beta cell function is moderately associated with (parts of) the risk for development of type 1 diabetes. Indeed, high GRS-1 and GRS-2 are both associated with lower C-peptide levels, and this association appears to show a strong inverse correlation with age of onset, while adult age of onset is specifically associated with more residual C-peptide production [12]. We therefore hypothesise that people with a higher GRS-1 and GRS-2 have a higher chance of more aggressive disease and therefore lower residual beta cell function. However, as the univariate correlations between residual UCPCR and GRS-1 and GRS-2 are only moderate, genetic risk may not fully explain long-term preservation of beta cell function [22].

We next evaluated the relationship between residual beta cell function and GRS-T2D, to assess whether a higher GRS-T2D results in a different disease progression [34]. We did not find a significant association between the GRS-T2D and residual beta cell function, although the direction of association was similar to a previously published association between higher type 2 diabetes genetic risk and higher random C-peptide levels [20, 21]. It may be that this association is only moderate, as there was an unadjusted correlation between higher GRS-T2D and higher UCPCR. However, it may also be that the type 2 diabetes genetic risk was not adequately captured by our GRS-T2D, as the imputation quality of almost half of the proposed SNPs was too low for them to be included in our score. Interestingly, a higher GRS-T2D was associated with lower TBR in unadjusted models. As TBR is a marker for hypoglycaemia, it may be that participants with higher GRS-T2D are more prone to insulin resistance, as this is a hallmark of type 2 diabetes [39]. This is supported by the fact that there is an association between higher GRS-T2D and lower GCV, which is a marker of glucose fluctuation. Moreover, in a subset analysis of participants with a longer duration of type 1 diabetes, the association remained significant in all models, and participants in previous studies who showed more severe insulin resistance had a higher GRS-T2D [24].

In a previous meta-GWAS, C-peptide production was found to be associated with multiple variants in the HLA region, as well as a locus on chromosome 1, that are not associated with type 1 diabetes itself. Some of the SNPs/loci related to type 1 or 2 diabetes also showed a relationship with C-peptide levels. We therefore formulated our own GRS based on SNPs that were significantly associated with random C-peptide levels in a recent Finnish study [20], combining those SNPs previously associated with type 1 and 2 diabetes and C-peptide levels. However, while we did observe that a higher GRS-C2 was significantly associated with detectable residual beta cell function after adjusting for age of onset and sex (Model 3), this did not persist after adjustment for duration of disease. This may indicate that the genetic contribution to maintaining residual beta cell function is only modest, and environmental factors play a more important role. However, we should keep in mind that the weights used in our GRS were based on random C-peptide levels in a Finnish population as a continues variable and, due to low imputation quality, not all significant SNPs (from the Finnish cohort) were included in our score. As this approach may have resulted in a less optimised score, in future investigations we aim to base the GRS-C1 and GRS-C2 on weights and SNPs derived from our own cohort, or even from a new GWAS on detectable UCPCR, and replicate our findings in a validation cohort.

### Strengths and limitations

Our study has several limitations. While UCPCR is a well-validated marker for stimulated C-peptide production in cohort studies, with an almost identical sensitivity and specificity to a mixed meal test [29], previous cohort studies mostly used random plasma C-peptide, which may have underestimated the observed association. Due to the relatively small sample size for this specific research question, there is a risk of the study being underpowered and underestimating the association between genetic risk and residual C-peptide production. Moreover, use of larger cohorts or meta-analyses may shed light on the observed sex differences in the associations between GRS and glycaemic control parameters. As aforementioned, missing genetic signalling data may have led to underestimation of the associations. Participants used their physician-prescribed CGM device for this study; therefore, the association with CGM metrics and genetic risk may be underestimated; however, we did not find a difference in CGM or pump use in the various tertiles of GRS-1. Lastly, as our inclusion criteria only allowed participants above 18 years old, it is difficult to truly separate the effect of longstanding type 1 diabetes duration from early age of onset, which should be addressed in future research.

### Conclusion

There is an association between higher genetic risk for type 1 diabetes and lower residual C-peptide production in type 1 diabetes. Furthermore, use of a newly developed GRS to assess C-peptide-specific genetic risk showed that a higher score was associated with detectable residual beta cell function, especially in participants with European genetic ancestry. However, none of the associations were significant after correction for age of onset and disease duration. Future meta-analyses and replication studies in larger samples are therefore warranted to further investigate the proportion of genetic contribution to maintaining residual beta cell function. Combining the genetic contribution with environmental triggers may increase precision prediction of maintenance of residual beta cell function and identification of promising therapeutic targets.

## Supplementary Information

Below is the link to the electronic supplementary material.

Supplementary file1 (PDF 1689 KB)

## Data Availability

The datasets produced and examined in the present study are not accessible to the public. However, they may be obtained from the corresponding author upon reasonable request.

## Abbreviations

CGM: Continuous glucose monitoring
GCV: Glucose coefficient of variance
GRS: Genetic risk score(s)
TAR: Time above range
TBR: Time below range
TIR: Time in range
UCPCR: Urinary C-peptide/creatinine ratio
