# Supplementary material for: Associations between diabetes-related genetic risk scores and residual beta cell function in type 1 diabetes: the GUTDM1 study
Source: Diabetologia. 2024 Jun 26;67(9):1865–76. doi: 10.1007/s00125-024-06204-6 (PMC11410997; doi:10.1007/s00125-024-06204-6)
Supplement: Supplementary file 1 — Supplementary file1 (PDF 1689 KB) [file 125_2024_6204_MOESM1_ESM.pdf]

ESM methods

Urinary C-Peptide to Creatinine ratios and glucagon measures

In order to evaluate the residual insulin production of participants, we measured stimulated urinary C-peptide/creatinine ratio (UCPCR) in nmol/mmol via electrochemiluminescence immunoassay, which was analyzed with the Roche P800 platform, as previously validated [1]. 2-hour post prandial UCPCR is a thoroughly validated marker for the quantification of residual C-peptide and serves as a highly effective non-invasive substitute for the more intrusive mixed meal test [2]. UCPCR remains stable in boric acid at room temperature [1]. Participants were characterized as UCPCR detectable when there was a UCPCR ≥ 0.01 nmol/mmol and as non-detectable when there was no UCPCR detectable. In 8 individuals we observed a C-peptide level that was just above the detection limit, but a calculated UCPCR below 0.01. We classified these individuals as non-detectable for conservative estimates of the associations and to avoid false positive cases.

Glucagon was measured in fasted plasma samples with a Mercodia human glucagon ELISA kit (10-1271-01).

CGM-metrics

All participants utilized either a real time CGM or, at a minimum, a second-generation intermittently scanned CGM (IS-CGM), as per the standard care provisions in the Netherlands. Prior to their study visit, participants gathered CGM data for a 14-day period, with a prerequisite sensor activation time at or above 90%. Time spend below range (TBR, <3.9mmol/l), in range (TIR, glucose concentrations of 3.9-10mmol/l), above range (TAR, >10mmol/l) and the glucose coefficient of variance (GCV), respectively, were calculated using the respective algorithms provided by the CGM manufacturers. These values were in

accordance with established guidelines [3]. The sensor's algorithm generated the GCV for glucose levels.

DNA isolation and imputation

Genotyping and Quality Control

To isolate DNA we used the QIAamp® DNA Mini Kit (250) (QIAGEN, Ref# 51306, Lot# 169049798) per protocol [4]. DNA was isolated from buffy coats, collected and stored in EDTA at -80°C, or PBMC samples (~1*10^6 cells in HI FBS/10% DMSO) [5]. SNPs were genotyped on the Infinium® ImmunoArray-24 v2.0 BeadChip (Illumina), at Erasmus MC, Rotterdam, the Netherlands. Variants were aligned to the positive strand with the WRayners strand alignment tool or resolved manually afterwards [6]. Duplicate SNPs were removed, keeping the ones with the lowest missingness rates. Sex-check was performed in PLINK 1.9 and potential duplicates were investigated using king 2.2.4 [7]. Samples with discrepancies in genetically determined sex and self-reported sex or identified as duplicates were selected for further investigation and excluded if this could not be resolved. Further SNP sample QC were performed in a two-step approach, first filtering out all SNPs with missingness > 0.2 and samples with missingness > 0.2, followed by a stricter quality control of missingness > 0.02 for both variant and sample missingness. Afterwards, SNPs with MAF < 0.05 were removed as well as SNPs out of Hardy-Weinberg equilibrium (p<1x10-6 in total cohort). Potential cryptic relatedness was investigated with king 2.2.4 [7], but no samples were considered to be 2nd degree relatives or closer relatives. Heterozygosity rate was checked using the Method-of-moments F coefficient estimate (F within +/-0.20), but no samples were removed.

Determining genetic ancestry

In order to determine genetic ancestry, we compared the GUTDM1 with the 1000 Genomes (1000G) dataset. Therefore, the quality controlled genotype data of the GUTDM1 cohort was processed in PLINK 1.9 by removing the HLA region and other regions with high linkage disequilibrium and pruning SNPs (--indep-pairwise 200 10 0.1) to obtain a set of variants that were not in linkage disequilibrium. Those SNPs (if present) were then extracted from the 1000G dataset and both datasets were merged after which additional quality control was performed. We used the GCTA (1.92.3) tool to perform a principal component analysis (PCA) on the autosomal SNPs of the 1000G dataset. Loadings of this PCA were used to project the GUTDM1 cohort on the 1000G cohort. The first 4 Principal Components were then used in kmeans analysis to label the genetic ancestry of the GUTDM1 cohort as either European (EUR), African (AFR), Ad Mixed American (AMR), East Asian (EAS) or South Asian (SAS) (ESM Figure 4).

Imputation

Quality controlled autosomal SNPs were uploaded to the TOPMed imputation server [8]. Variants were imputed with the Quality Control & Imputation mode, using TOPMed r2 as reference panel, Eagle v2.4 for phasing and vs. TOPMED Panel as population. The TOPMed Imputation Server automatically updates the genome positions of the variants from GRCh37 to GRCh38. Variants with imputation quality ($R2$) < 0.7 were removed with bcftools (1.10.2). Remaining variants were converted to hard calls in PLINK format.

References

1. McDonald, T.J., et al., *Stability and reproducibility of a single-sample urinary C-peptide/creatinine ratio and its correlation with 24-h urinary C-peptide.* Clin Chem, 2009. **55**(11): p. 2035-9.
2. Besser, R.E., et al., *Urine C-peptide creatinine ratio is a noninvasive alternative to the mixed-meal tolerance test in children and adults with type 1 diabetes.* Diabetes Care, 2011. **34**(3): p. 607-9.
3. Battelino, T., et al., *Clinical Targets for Continuous Glucose Monitoring Data Interpretation: Recommendations From the International Consensus on Time in Range.* Diabetes Care, 2019. **42**(8): p. 1593-1603.
4. van der Vossen, E.W.J., et al., *Effects of fecal microbiota transplant on DNA methylation in subjects with metabolic syndrome.* Gut Microbes, 2021. **13**(1): p. 1993513.
5. Fuhri Snethlage, C.M., et al., *Residual β-Cell Function Is Associated With Longer Time in Range in Individuals With Type 1 Diabetes.* Diabetes Care, 2023.
6. Rayner, N. and M. McCarthy. *Development and use of a pipeline to generate strand and position information for common genotyping chips*. in *Annual Meeting of the American Society of Human Genetics, Montreal, Canada*. 2011.
7. Manichaikul, A., et al., *Robust relationship inference in genome-wide association studies.* Bioinformatics, 2010. **26**(22): p. 2867-73.
8. Das, S., et al., *Next-generation genotype imputation service and methods.* Nature Genetics, 2016. **48**(10): p. 1284-1287.

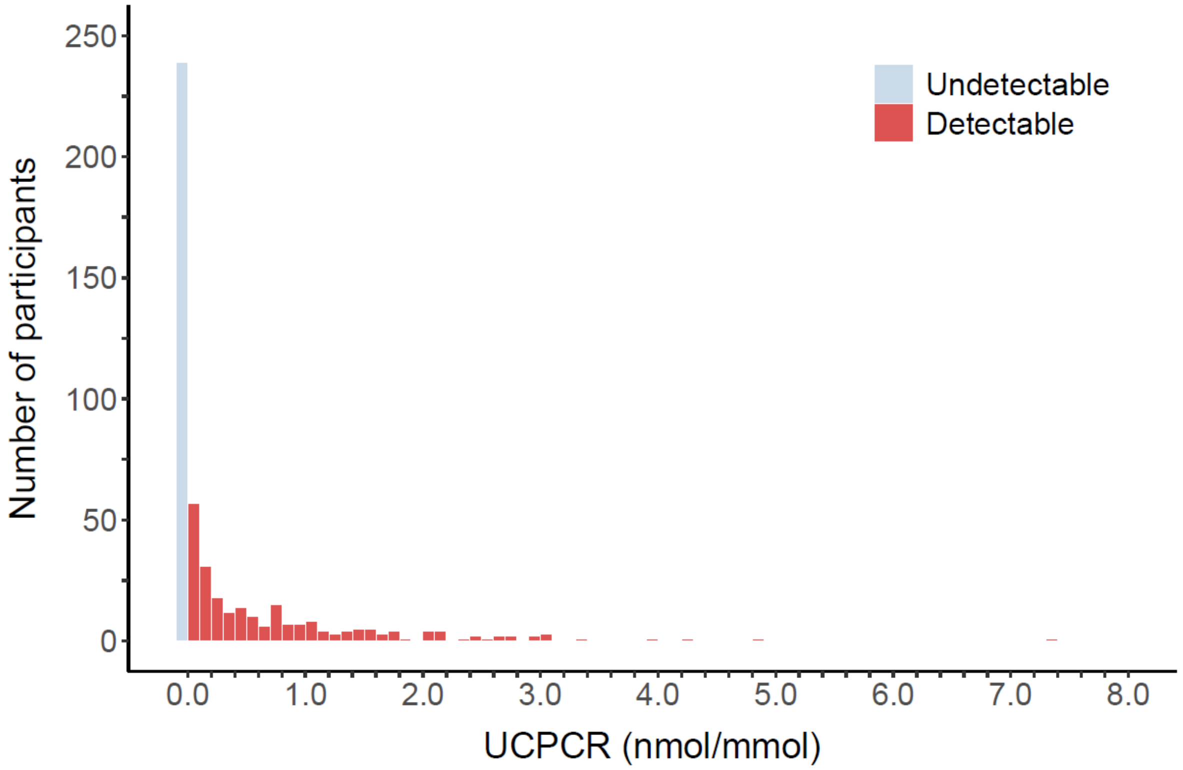

ESM Figure 1 Distribution histogram of UCPCR, with undetectable in blue and detectable in red.

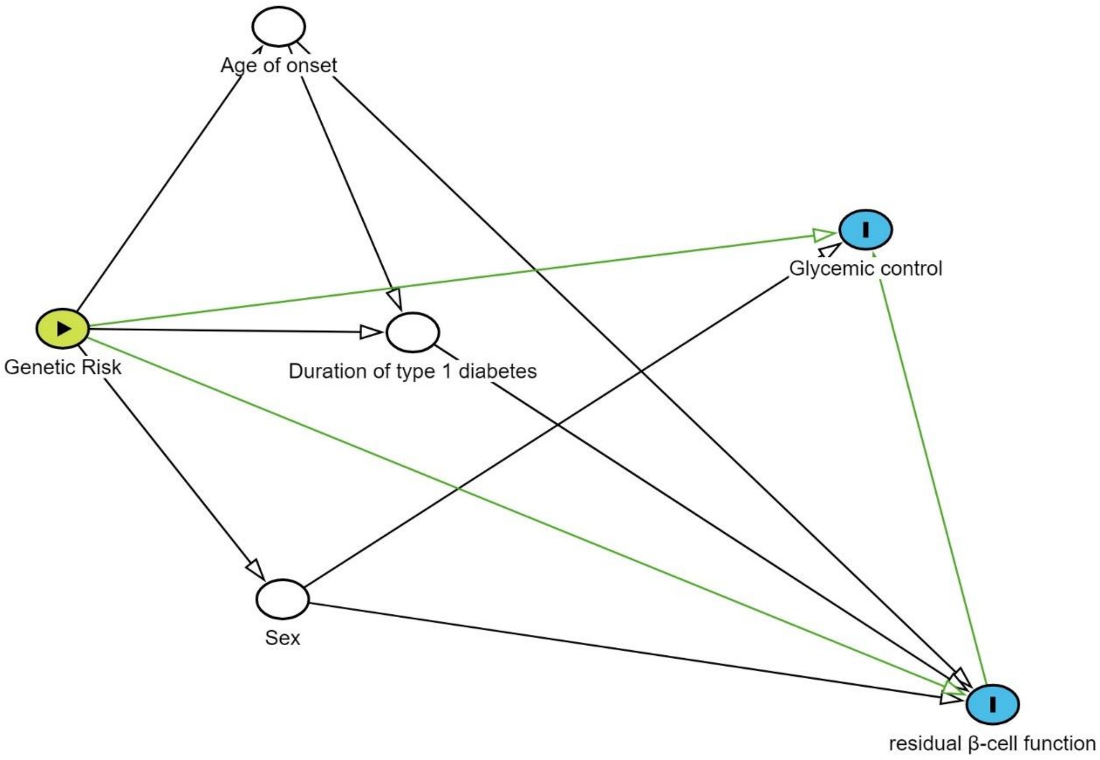

ESM Figure 2 A directed acyclic graph was added to show potential biasing pathways, cofounding and correction for cofounders. Models were adjusted for sex, age of onset and duration of disease

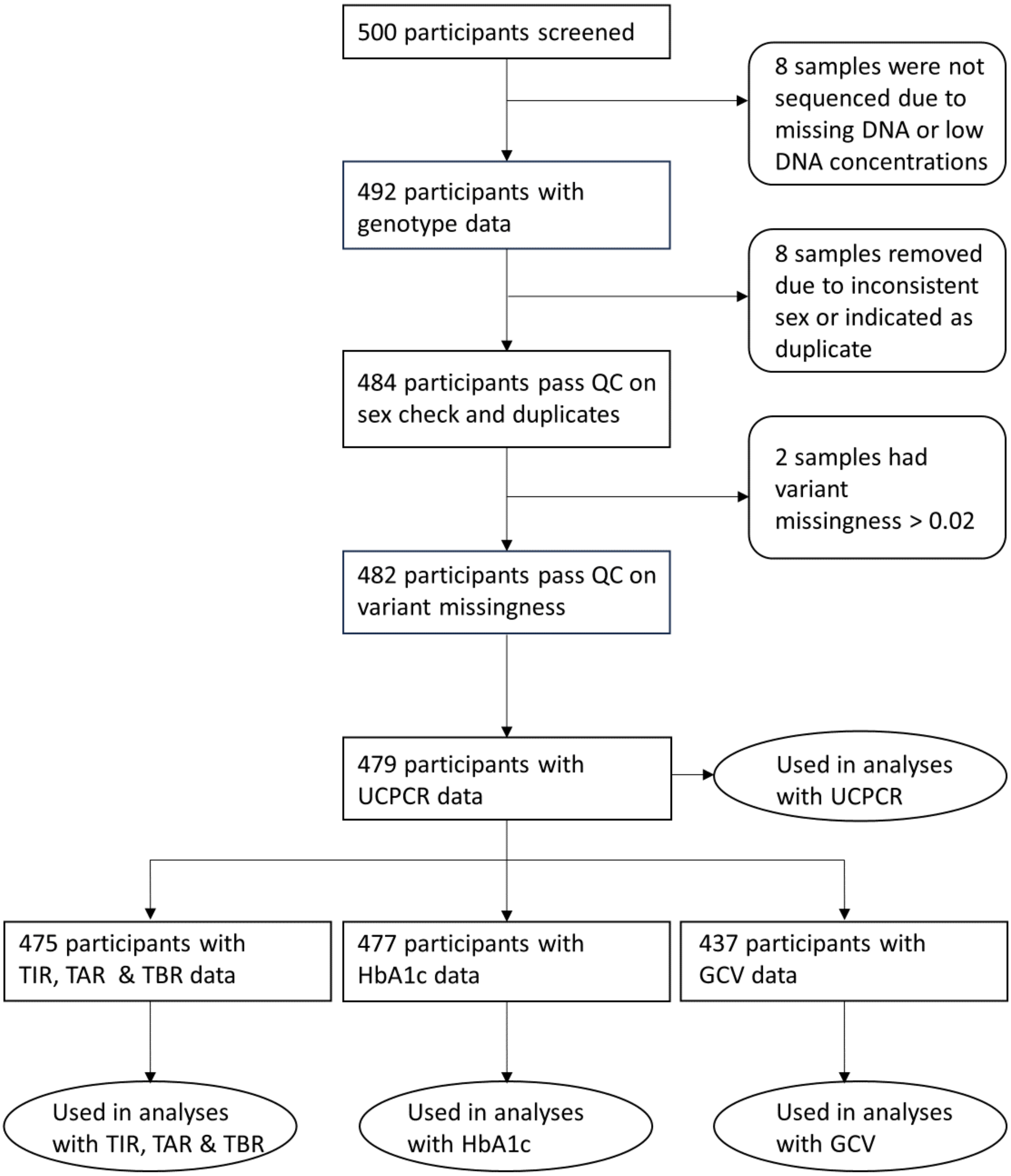

ESM Figure 3 Inclusion flowchart *479 Participants had GRS and UCPCR data and were included in the main analyses. Of these participants, 475 had time in range, time above range, time below range data available. 477 had* HbA_{1c} *data available and 437 had GCV data available and were included in the analysis with genetic risk.*

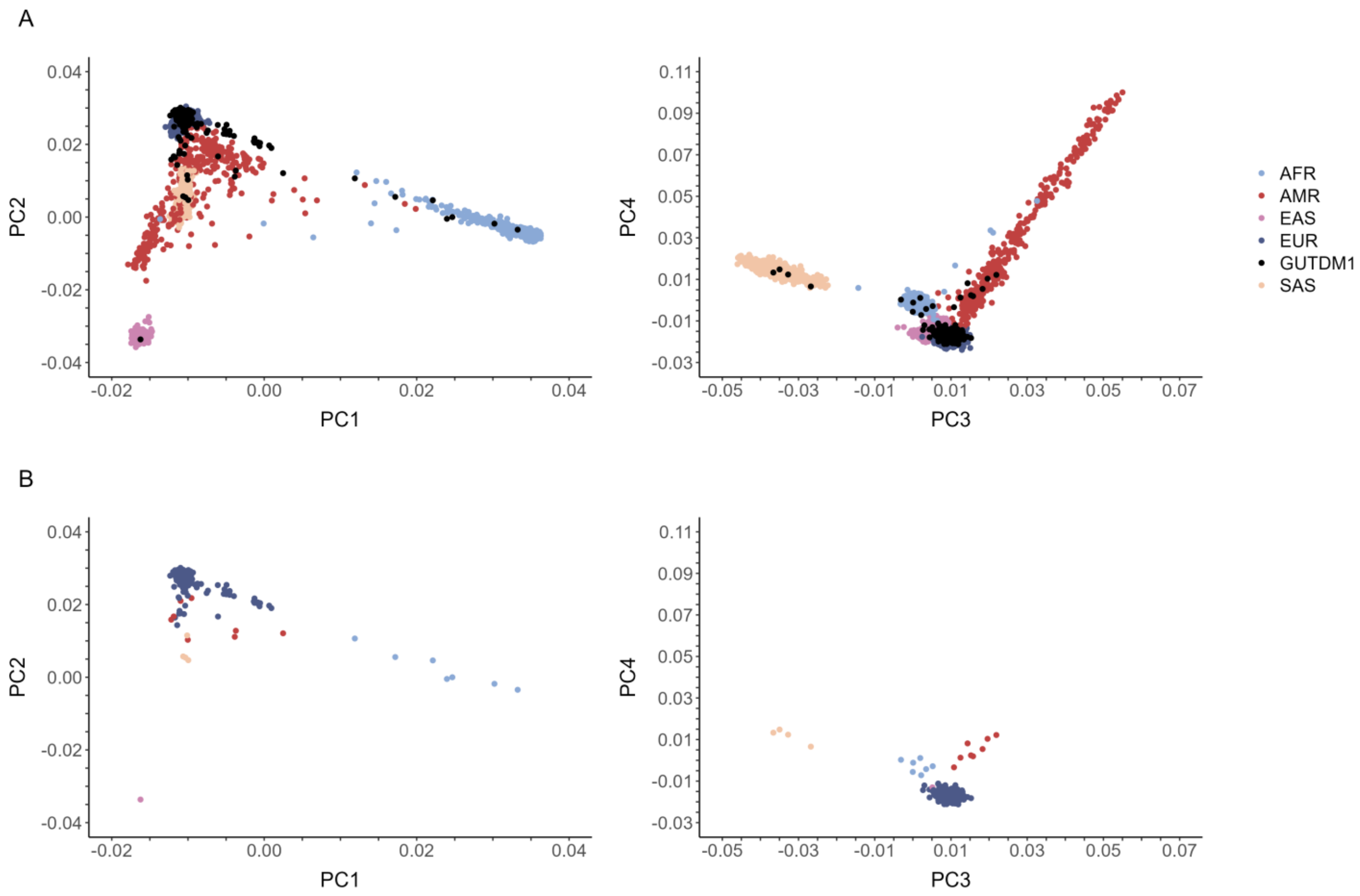

ESM Figure 4 *Determination of genetic ancestry of GUTDM1. GUTDM1 samples (n=482) were projected onto the 1000Genomes dataset (A) and assigned a genetic ancestry based on kmeans clustering on the first four principle components (B). AFR = African, AMR = Ad Mixed American, EAS = East Asian, EUR = European, SAS = South Asian.*

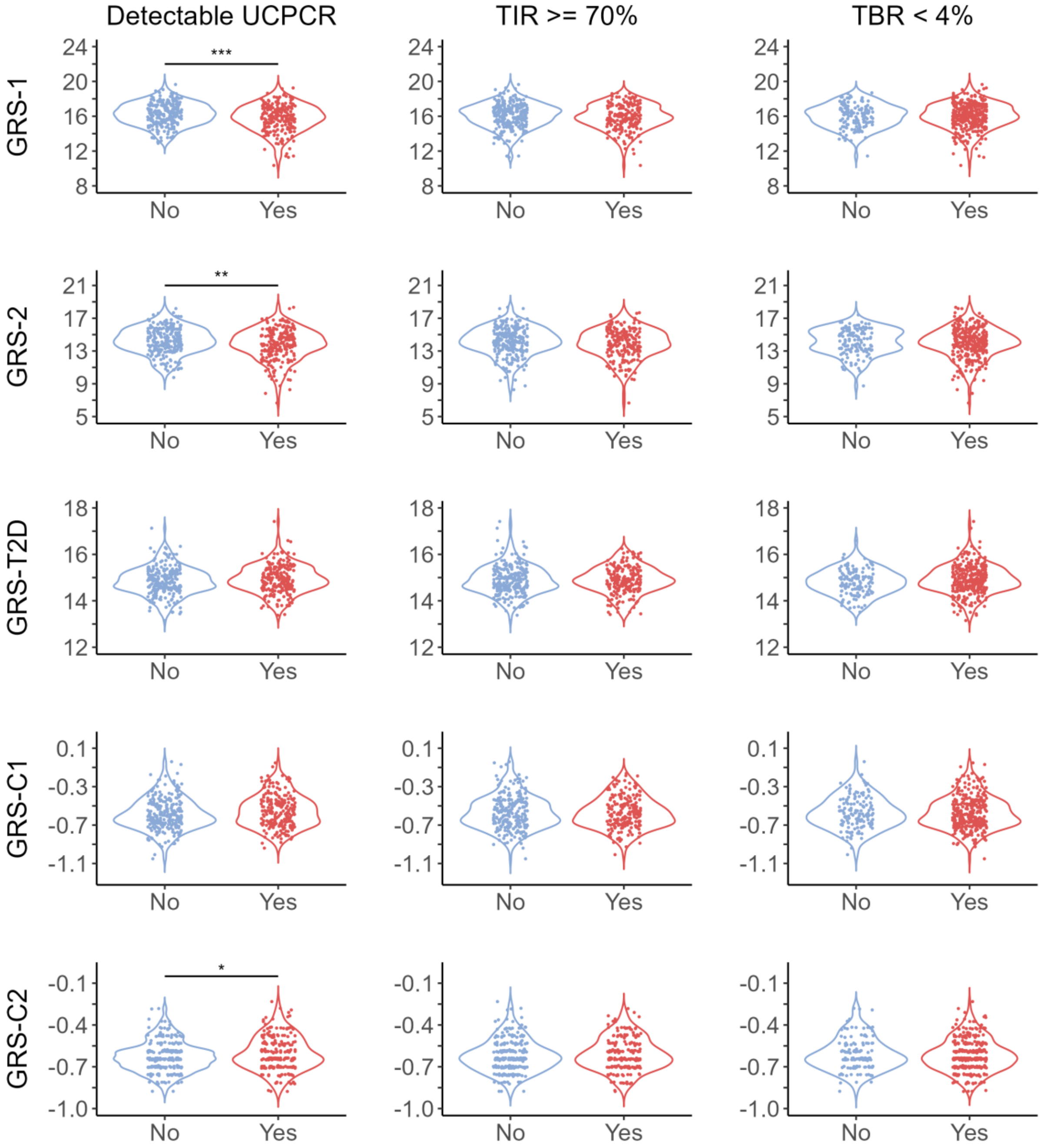

ESM Figure 5 *Violin plot visualization of all GRS in only people with European genetic ancestry. If participants had a detectable UCPCR they were classified as yes, otherwise classified as no. Time in range in percentage ≥70% or Time below range was < 4% was classified as yes, otherwise no. Significant differences between groups were tested with a t-test and indicated with *** (p-value <0.001), ** (p-value < 0.01) and * (p-value <0.05)*

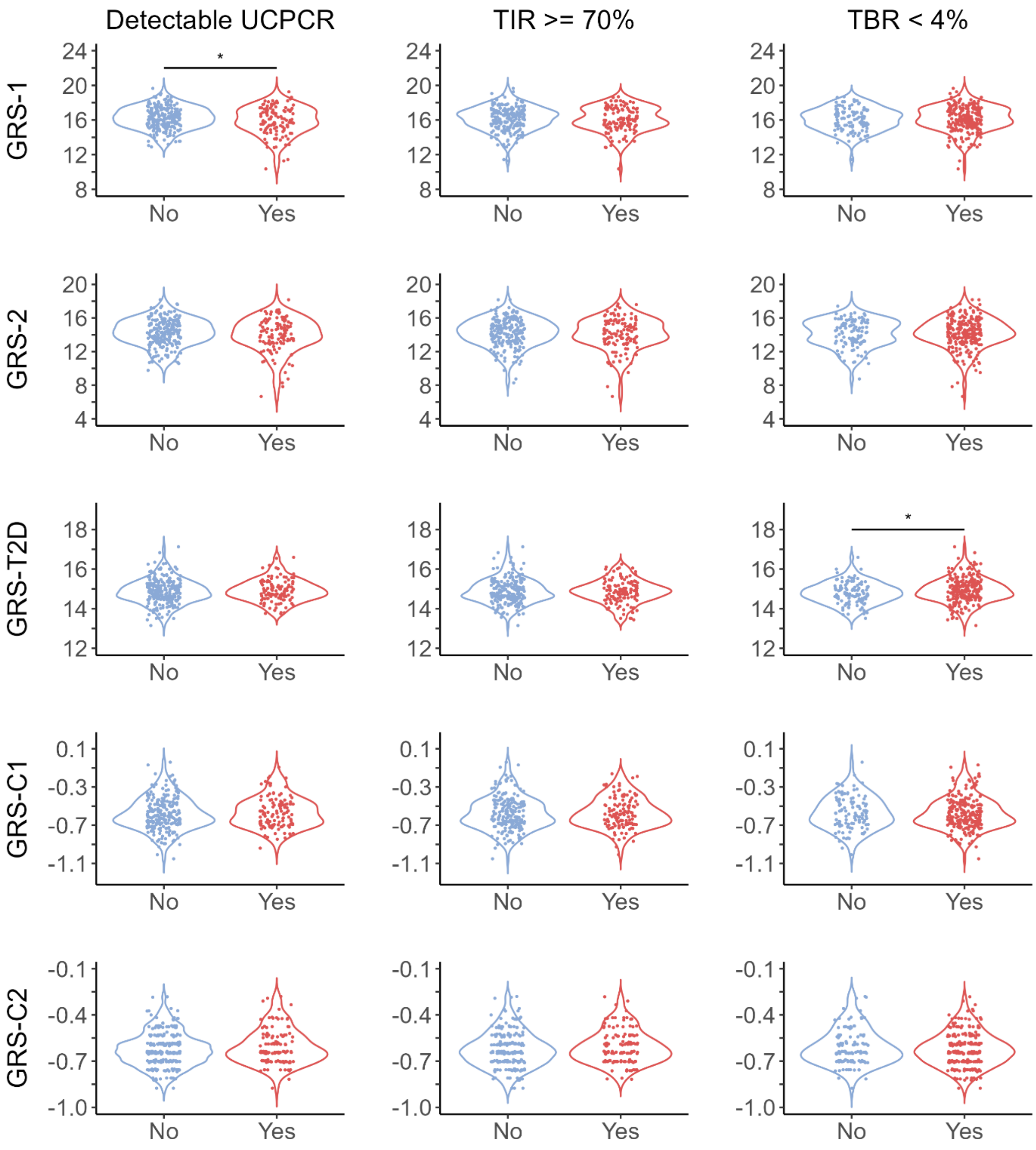

ESM Figure 6 *Violin plot visualization of all GRS in all participants with duration of type 1 diabetes more than 7 years. If participants had a detectable UCPCR they were classified as yes, otherwise classified as no. Time in range in percentage ≥70% or Time below range was < 4% was classified as yes, otherwise no. Significant differences between groups were tested with a t-test and indicated with *** (p-value <0.001), ** (p-value < 0.01) and * (p-value <0.05).*

ESM Table 1 *SNPs used in the generation of GRS-1. Position of the SNP is based on build GRCh38 on the positive strand. The effect alleles and corresponding weights were used in the calculations.*

Gene	POS (GRCh38)	REF	ALT	Rsid	Effect allele	Weight
DR3	6:32658707	T	C	rs9273369	C	Combined
DR4-DQ8	6:32713706	T	C	rs7454108	C	Combined
HLA_A_24	6:29972123	C	T	rs1264813	T	0.43
HLA_B_5701	6:31464003	T	G	rs2395029	T	0.92
HLA_DRB1_15	6:32445768	G	A	rs3129889	A	2.70
PTPN22	1:113834946	A	G	rs2476601	A	0.67
INS	11:2160994	A	T	rs689	T	0.56
IL2RA	10:6055320	T	C	rs12722495	T	0.46
ERBB3	12:56088396	T	G	rs2292239	T	0.30
C10orf59	10:88263276	T	C	rs10509540	T	0.29
COBL	7:50959497	A	C	rs4948088	C	0.26
	16:75213347	T	G	rs7202877	G	0.25
CLEC16A	16:11086016	A	G	rs12708716	A	0.21
CTLA4	2:203874196	G	A	rs3087243	G	0.20
PTPN2	18:12809341	A	G	rs1893217	G	0.18
IL2RA	10:6080046	T	A	rs11594656	T	0.17
IL10	1:206766559	G	A	rs3024505	G	0.17
C6orf173	6:126377573	A	G	rs9388489	G	0.16
	14:68796882	T	C	rs1465788	C	0.15
IFIH1	2:162267541	C	T	rs1990760	T	0.15
CTSH	15:78943104	T	C	rs3825932	C	0.15
	19:46705224	T	C	rs425105	T	0.15
CD226	18:69864406	T	C	rs763361	T	0.15
IL27	16:28528527	C	T	rs4788084	C	0.15
	15:38610275	A	C	rs17574546	C	0.13
BACH2	6:90248512	C	G	rs11755527	G	0.12
UBASH3A	21:42421219	C	A	rs3788013	A	0.12
IL2	4:122456825	A	C	rs2069762	A	0.11
	20:1629905	T	C	rs2281808	C	0.10
	22:30185733	C	T	rs5753037	T	0.10

ESM Table 2 *Weights to use in DR-DQ score of GRS-1. Specific SNP and effect allele for DR3 and DR4-DQ8 are present in Supplementary Table 1. X = nonDR3 and nonDR4-DQ8.*

Allele2	Allele1	Weight
DR3	DR4-DQ8	3.87
DR3	DR3	3.05
DR4-DQ8	DR4-DQ8	3.09
DR4-DQ8	X	1.95
DR3	X	1.51

ESM Table 3 *SNPs used in the generation of GRS-2. Position of the SNP is based on build GRCh38 on the positive strand. The effect alleles and corresponding weights were used in the calculations. Weights for the HLA-DQ haplotypes are provided in Supplementary Table 5 and 6.*

SNP type	Gene/locus	POS (GRCh38)	REF	ALT	Rsid	Effect allele	Weight
HLA-DQ	HLA-DQ81	6:32705608	C	G	rs9275490	G	NA
HLA-DQ	HLA-DQ62	6:32644083	A	G	rs9273032	A	NA
HLA-DQ	HLA-DQ25	6:32658707	T	C	rs9273369	C	NA
HLA-DQ	HLA-DQ22	6:32658260	G	T	rs17211699	T	NA
HLA-DQ	HLA-DQ75	6:32635435	T	C	rs9469200	C	NA
HLA-DQ	HLA-DQ51	6:32709663	G	A	rs10947332	A	NA
HLA-DQ	HLA-DQ73	6:32616043	G	T	rs1281935	T	NA
HLA-DQ	HLA-DQ63	6:32704437	G	T	rs62406889	T	NA
HLA-DQ	HLA-DQ92	6:32680817	A	G	rs28746898	G	NA
HLA-DQ	HLA-DQ42	6:32712215	C	T	rs12527228	T	NA
HLA-DQ	HLA-DQ53	6:32706960	C	G	rs1794265	G	NA
HLA-DQ	HLA-DQ93	6:32634974	C	A	rs9405117	A	NA
HLA-DQ	HLA-DQ69	6:32415221	G	C	rs17840116	C	NA
HLA-DQ	HLA-DQ61	6:32658670	G	A	rs117806464	A	NA
HLA-other	B*3906	6:31307016	G	GC	rs540653847	GC	1.78
HLA-other	XL9 Regulatory	6:32615766	A	G	rs9271347	G	1.69
HLA-other	BTNL2 Regulatory	6:32408740	C	T	rs9268500	C	1.24
HLA-other	Intergenic DRB1-DQA1	6:32630313	T	C	rs1281943	C	0.9
HLA-other	DPB1*1501	6:33081408	G	A	rs2567287	A	0.84
HLA-other	Intergenic BTNL2-DRA1	6:32427740	T	C	rs75658393	T	0.81
HLA-other	A*2402	6:29868825	C	T	rs72848653	T	0.78
HLA-other	A*2902	6:29840255	C	G	rs1233320	G	0.74
HLA-other	Intergenic DRA1-DRB1	6:32479411	T	A	rs9269173	A	0.67
HLA-other	A*0205	6:29760476	G	T	rs9500974	T	0.63
HLA-other	C*0602	6:31284147	C	T	rs12189871	T	0.45
HLA-other	A*0201	6:29946312	G	A	rs12153924	A	0.44
HLA-other	B*1801	6:31358273	G	A	rs9266268	A	0.39
HLA-other	A*0301	6:29883132	T	C	rs9259118	T	0.31
HLA-other	DPB1*0301	6:33103130	G	A	rs3129197	A	0.24
HLA-other	DPB1*0101	6:33079396	T	C	rs17214657	C	-0.19
HLA-other	DPB1*0501	6:33081532	A	G	rs9378176	G	-0.49
HLA-other	B*4403	6:31439802	G	A	rs2524277	A	-0.6
HLA-other	DPB1*0402	6:33077179	T	C	rs6934289	C	-0.68
HLA-other	B*4501	6:31375490	G	A	rs16899379	A	-0.83

HLA-other	B*5701	6:31376405	TG	T	rs149663102	T	-0.94
Non-HLA	INS	11:2159830	T	G	rs3842753	G	0.83
Non-HLA	PTPN22	1:113761186	C	A	rs6679677	A	0.64
Non-HLA	CTSH	15:78944951	C	T	rs2289702	C	0.28
Non-HLA	ATXN2	12:111569952	C	T	rs653178	C	0.26
Non-HLA	COBL	7:50959497	A	C	rs4948088	C	0.26
Non-HLA	IL27	16:28580209	G	A	rs9924471	A	0.22
Non-HLA	ERBB3	12:56080696	A	G	rs4759229	A	0.22
Non-HLA	PTPN2	18:12809341	A	G	rs1893217	G	0.19
Non-HLA	BACH2	6:90267049	G	A	rs72928038	A	0.18
Non-HLA	RNLS	10:88291560	A	G	rs60888743	A	0.18
Non-HLA	ITGB7	12:53192075	C	T	rs11170466	T	0.17
Non-HLA	UBASH3A	21:42405613	G	C	rs9981624	C	0.17
Non-HLA	CENPW	6:126377573	A	G	rs9388489	G	0.16
Non-HLA	HORMAD2	22:30108663	A	G	rs5763779	A	0.15
Non-HLA	PRKD2	19:46705224	T	C	rs425105	T	0.15
Non-HLA	RASGRP1	15:38554821	C	T	rs72727394	T	0.14
Non-HLA	ADAD1	4:122408207	G	A	rs17388568	A	0.12
Non-HLA	CD226	18:69859408	T	C	rs1615504	T	0.12
Non-HLA	GLIS3	9:4290823	A	T	rs6476839	T	0.11
Non-HLA	IRF7	13:99429512	C	T	rs9585056	C	0.11
Non-HLA	C1QTNF6	22:37195278	G	A	rs229541	A	0.1
Non-HLA	SIRPG	20:1629905	T	C	rs2281808	C	0.1
Non-HLA	TAGAP	6:159044945	T	C	rs1738074	T	-0.08
Non-HLA	MEG3	14:100840110	T	C	rs56994090	C	-0.13
Non-HLA	CLEC1	12:9733403	G	A	rs10492166	A	-0.14
Non-HLA	IL10	1:206766559	G	A	rs3024505	A	-0.15
Non-HLA	IFIH1	2:162254026	A	G	rs2111485	A	-0.16
Non-HLA	CTLA4	2:203874196	G	A	rs3087243	A	-0.17
Non-HLA	CLEC16A	16:11086016	A	G	rs12708716	G	-0.19
Non-HLA	TYK2	19:10381598	C	T	rs144309607	T	-0.4
Non-HLA	IL2RA	10:6052734	C	T	rs61839660	T	-0.48
Non-HLA	IL2RA	10:6087680	C	T	rs41295121	T	-0.71

ESM Table 4 *Ranks to use when more than 2 DQ haplotypes are present in GRS-2.*

Haplotypes should be selected based on the highest rank (i.e. 1 is higher than 2).

**DQ**	**Rank**
DQ62	1
DQ25	2
DQ51	3
DQ22	4
DQ75	5
DQ81	6
DQ63	7
DQ73	8
DQ92	9
DQ64	10
DQ42	11
DQ53	12
DQ52	13
DQ93	14
DQ69	15
DQ61	16
DQ76	17

ESM Table 5 *Weight for the DR-DQ score in GRS-2, in case both HLA haplotypes show an interaction. Corresponding SNPs and effect alleles are listed in Supplementary Table 3.*

**Haplotype 1**	**Haplotype 2**	**Weight**
DQ25	DQ81	3.632269
DQ73	DQ51	0.054796
DQ81	DQ81	3.135265
DQ73	DQ81	0.171785
DQ25	DQ73	-0.51343
DQ73	DQ22	-0.69142
DQ42	DQ22	0.610013
DQ25	DQ51	1.064822
DQ25	DQ22	0.17115
DQ25	DQ25	2.163682
DQ62	DQ62	-0.23695
DQ25	DQ75	-0.6538
DQ25	DQ63	-0.87311
DQ81	DQ63	1.08404
DQ73	DQ73	-1.15482
DQ93	DQ81	2.308103
DQ73	DQ75	-1.46845
DQ25	DQ62	-1.93572

ESM Table 6 *Weights for the DR-DQ score in GRS-2, in case both HLA haplotypes did not show an interaction. Corresponding SNPs and effect alleles are listed in Supplementary Table 3.*

**DQ**	**Weight**
DQ81	2.078323
DQ25	1.262934
DQ93	1.391622
DQ42	-0.46092
DQ51	-0.02948
DQ73	-0.64577
DQ22	-0.75371
DQ63	-2.21639
DQ69	-0.62952
DQ75	-1.32274
DQ61	0.479641
DQ92	-2.35528
DQ53	-0.88502
DQ62	-1.42689

ESM Table 7 *SNPs used in the generation of GRS-T2D. Position of the SNP is based on build GRCh38 on the positive strand. The effect alleles and corresponding weights were used in the calculations. The gene listed is nearest gene.*

Gene	POS (GRCh38)	REF	ALT	rsID	Effect allele	Weight
MACF1	1:39570256	G	T	rs3768321	T	0.0861777
FAF1	1:50790419	C	T	rs58432198	C	0.06765865
PATJ	1:62114219	G	T	rs12140153	G	0.06765865
DENND2C	1:114602278	T	C	rs184660829	C	2.08567209
PTGFRN	1:116990168	C	T	rs1127215	C	0.04879016
NOTCH2	1:119984359	C	T	rs1493694	T	0.0861777
FAM63A	1:151045515	T	C	rs145904381	T	0.17395331
SEC16B	1:177919890	A	C	rs539515	C	0.04879016
DSTYK	1:205145745	C	G	rs12048743	G	0.03922071
SRGAP2	1:206420549	G	C	rs9430095	C	0.03922071
PROX1	1:213977478	G	C	rs79687284	C	0.14842001
PROX1	1:213985913	T	C	rs340874	C	0.06765865
PROX1	1:214002188	T	G	rs114526150	G	0.11332869
LYPLAL1	1:219575476	C	G	rs2820446	C	0.05826891
TMEM18	2:653575	C	T	rs35913461	C	0.05826891
DTNB	2:25420352	G	T	rs17802463	G	0.03922071
GCKR	2:27508073	T	C	rs1260326	C	0.06765865
THADA	2:42980732	G	T	rs28525376	G	0.0295588
THADA	2:43203301	G	A	rs6708643	A	0.03922071
THADA	2:43470889	A	T	rs80147536	A	0.12221763
BNIPL	2:58753929	G	T	rs10193538	T	0.03922071
BCL11A	2:60356530	G	A	rs243024	A	0.05826891
CEP68	2:65427878	C	G	rs2028150	C	0.04879016
DDX18	2:117313485	A	G	rs562386202	G	1.16315081
PABPC1P2	2:147104065	C	T	rs35999103	T	0.04879016
CYTIP	2:157483038	A	G	rs13426680	A	0.0861777
RBMS1	2:160279033	C	T	rs3772071	T	0.04879016
GRB14/COBLL1	2:164656581	T	C	rs10195252	T	0.06765865
GRB14/COBLL1	2:164716684	C	T	rs13024606	T	0.0861777
IRS1	2:226236695	A	G	rs2972144	G	0.09531018
PPARG	3:12295008	G	A	rs11709077	G	0.13102826
PPARG	3:12447843	T	G	rs17819328	G	0.05826891
UBE2E2	3:23414091	T	C	rs35352848	T	0.06765865
UBE2E2	3:23468553	A	G	rs17013314	G	0.10436002
KIF9	3:46884049	T	C	rs11926707	C	0.2390169
KIF9	3:47201433	A	G	rs75423501	G	0.04879016
RBM6	3:49943163	C	T	rs4688760	T	0.03922071
RFT1	3:53093661	G	T	rs2581787	T	0.03922071
CACNA2D3	3:54794800	G	T	rs76263492	T	0.0861777

ADAMTS9	3:64715470	A	G	rs9860730	A	0.05826891
SHQ1	3:72816032	C	T	rs13085136	C	0.07696104
ADCY5	3:123346931	A	G	rs11708067	A	0.0861777
MBNL1	3:152700092	C	A	rs74653713	C	0.09531018
MBNL1	3:152715839	C	T	rs35497231	C	0.03922071
SLC2A2	3:171015287	G	A	rs9873618	G	0.06765865
ABCC5	3:184020672	A	C	rs2872246	A	0.03922071
IGF2BP2	3:185785668	T	A	rs6780171	A	0.13102826
IGF2BP2	3:185823425	G	T	rs11717959	G	0.03922071
IGF2BP2	3:186112102	T	A	rs1516728	A	0.0295588
LPP	3:188023111	T	C	rs4686471	C	0.05826891
PCGF3	4:751184	G	T	rs1531583	T	0.12221763
PCGF3	4:1016289	T	C	rs35654957	C	0.0295588
WFS1	4:6300792	G	A	rs1801212	A	0.04879016
WFS1	4:6305036	G	C	rs10937721	C	0.05826891
LCORL	4:17791246	A	C	rs12640250	C	0.03922071
GNPDA2	4:45184122	G	A	rs10938398	A	0.04879016
FAM13A	4:88819743	C	G	rs1903002	G	0.03922071
FAM13A	4:88936140	C	T	rs576406049	T	0.50077529
SMARCAD1	4:94170760	A	G	rs6821438	A	0.03922071
SLC9B1	4:103219691	C	A	rs1580278	C	0.03922071
TMEM154	4:152592217	T	A	rs7669833	T	0.05826891
ITGA1	5:52495391	T	C	rs17261179	T	0.03922071
ITGA1	5:52804655	A	G	rs3811978	G	0.05826891
ITGA1	5:53019852	G	A	rs62357230	A	0.0861777
ARL15	5:53478680	G	A	rs62370480	A	0.03922071
ARL15	5:53975590	G	A	rs702634	A	0.04879016
ARL15	5:54116790	C	A	rs279744	C	0.03922071
ANKRD55	5:56512648	C	T	rs465002	T	0.10436002
ANKRD55	5:56900777	G	A	rs96844	G	0.03922071
POC5	5:75707853	T	C	rs2307111	T	0.04879016
RASA1	5:87281535	A	G	rs7719891	G	0.03922071
PAM	5:103087264	C	T	rs115505614	T	0.17395331
PHF15	5:134528909	G	A	rs329122	A	0.03922071
EBF1	5:158501188	T	C	rs3934712	C	0.04879016
RREB1	6:7035501	C	A	rs112498319	C	0.0295588
RREB1	6:7255417	C	T	rs9505097	C	0.04879016
CDKAL1	6:20679478	A	G	rs7756992	G	0.13976194
MHC	6:32605638	A	G	rs601945	G	0.05826891
HMGA1	6:34279270	C	T	rs77136196	T	0.10436002
HMGA1	6:34556921	C	T	rs2233632	T	0.03922071
VEGFA	6:43792590	C	G	rs11967262	G	0.03922071
TFAP2B	6:50821065	A	C	rs3798519	C	0.05826891
TFAP2B	6:51315967	G	A	rs2465043	G	0.0295588
CENPW	6:126470949	A	G	rs11759026	G	0.06765865
SOGA3	6:127095785	A	G	rs2800733	A	0.04879016

MIR3668	6:139514192	A	T	rs2982521	A	0.04879016
SLC22A3	6:160349280	A	G	rs474513	A	0.03922071
DGKB	7:14858657	C	T	rs17168486	T	0.06765865
DGKB	7:15023944	C	T	rs10228066	T	0.06765865
IGF2BP3	7:23473277	G	C	rs4279506	G	0.05826891
JAZF1	7:28159058	C	T	rs1708302	C	0.09531018
GCK	7:44216044	G	A	rs878521	A	0.05826891
FBXL13	7:102845807	T	C	rs11496066	T	0.07696104
RELN	7:103804531	C	T	rs39328	T	0.03922071
CTTNBP2	7:117855613	C	A	rs6976111	A	0.03922071
KLF14	7:130387196	A	C	rs2268382	C	0.0295588
AOC1	7:150840547	G	A	rs62492368	A	0.04879016
MSRA	8:10117314	G	A	rs17689007	G	0.03922071
XKR6	8:10951177	A	T	rs57327348	A	0.03922071
LPL	8:19973410	C	T	rs10096633	C	0.06765865
PURG	8:31006422	T	C	rs10954772	T	0.03922071
ANK1	8:41651058	C	A	rs13262861	C	0.06765865
ANK1	8:41652396	C	T	rs4736819	T	0.03922071
TP53INP1	8:94949398	T	C	rs10097617	T	0.03922071
CPQ	8:96725513	G	A	rs149364428	A	0.2390169
TRHR	8:109110954	C	G	rs12680028	C	0.03922071
SLC30A8	8:117172786	G	A	rs3802177	G	0.10436002
CASC11	8:127699497	G	A	rs17772814	G	0.07696104
PVT1	8:128555832	C	T	rs1561927	C	0.03922071
BOP1	8:144654498	T	C	rs12719778	T	0.03922071
GLIS3	9:3965689	C	A	rs510807	A	0.0295588
GLIS3	9:4243045	T	A	rs79103584	T	0.13102826
GLIS3	9:4291928	A	C	rs10974438	C	0.04879016
HAUS6	9:19067835	A	G	rs7022807	G	0.03922071
FOCAD	9:20241071	T	C	rs7867635	C	0.03922071
CDKN2A/B	9:22043613	C	T	rs1412830	C	0.03922071
CDKN2A/B	9:22134069	G	A	rs10811660	G	0.2390169
LINGO2	9:28410685	T	C	rs1412234	C	0.03922071
UBAP2	9:34074478	T	C	rs12001437	C	0.03922071
MTND2P8	9:78744197	C	G	rs11137820	C	0.03922071
TLE4	9:79290675	A	G	rs17791513	A	0.09531018
ZNF169	9:94239400	A	C	rs55653563	A	0.03922071
ZNF169	9:94735212	T	C	rs12236906	T	0.13976194
ABO	9:133273813	C	T	rs505922	C	0.04879016
GPSM1	9:136341149	G	C	rs78403475	G	0.05826891
GPSM1	9:136346577	G	A	rs28505901	G	0.0861777
NEUROG3	10:69561523	A	G	rs177045	G	0.06765865
NEUROG3	10:69706822	T	G	rs2642588	G	0.04879016
ZMIZ1	10:79193069	G	C	rs703972	G	0.06765865
ZMIZ1	10:79336832	G	A	rs1317617	G	0.03922071
PTEN	10:88009583	C	T	rs11202627	T	0.05826891

HHEX/IDE	10:92702670	T	C	rs10882101	T	0.05826891
HHEX/IDE	10:92719350	A	G	rs1112718	A	0.05826891
TCF7L2	10:112998590	C	T	rs7903146	T	0.31481074
PLEKHA1	10:122433665	T	G	rs2280141	T	0.04879016
INS/IGF2	11:1683366	G	A	rs12802972	A	0.0295588
INS/IGF2	11:2097630	T	G	rs11042596	G	0.03922071
INS/IGF2	11:2176056	A	G	rs4929965	A	0.06765865
PDE3B	11:14742282	C	A	rs141521721	A	0.12221763
KCNJ11	11:17386857	C	T	rs5213	C	0.06765865
QSER1	11:32439327	C	T	rs7943101	T	0.03922071
PDHX	11:34960601	C	A	rs2767036	C	0.03922071
HSD17B12	11:43856384	C	A	rs1061810	A	0.04879016
CRY2	11:45890462	A	G	rs7115753	A	0.03922071
CELF1	11:47508395	C	A	rs7124681	A	0.03922071
MAP3K11	11:65527328	C	T	rs1783541	T	0.05826891
CCND1	11:69229758	G	A	rs61881115	G	0.04879016
CENTD2/ARAP 1	11:72749353	A	C	rs77464186	A	0.10436002
MTNR1B	11:92975544	C	G	rs10830963	G	0.09531018
ETS1	11:128364249	A	G	rs10750397	A	0.04879016
ETS1	11:128529043	C	T	rs67232546	T	0.05826891
ETS1	11:128714080	C	T	rs112595469	T	0.09531018
CCND2	12:3921938	C	T	rs10848958	C	0.03922071
CCND2	12:4275530	C	T	rs3217792	C	0.11332869
ITPR2	12:26300350	A	G	rs718314	G	0.04879016
HMGA2	12:65827280	A	T	rs2258238	T	0.09531018
HMGA2	12:65964567	C	T	rs1042725	T	0.04879016
TSPAN8/LGR5	12:71129173	G	C	rs1796330	G	0.04879016
RMST	12:97454997	A	G	rs77864822	A	0.07696104
WSCD2	12:108236003	G	A	rs1426371	G	0.04879016
KSR2	12:117974568	G	A	rs34965774	A	0.05826891
HNF1A	12:120860012	G	A	rs11065299	A	0.05826891
HNF1A	12:120942738	G	A	rs73226260	G	0.12221763
HNF1A	12:120979061	C	T	rs1800574	T	0.13102826
HNF1A	12:120994314	G	C	rs56348580	G	0.04879016
HNF1A	12:121063658	C	A	rs28638142	A	0.07696104
MPHOSPH9	12:122966218	G	C	rs4148856	C	0.04879016
ZNF664	12:124024630	G	A	rs825452	A	0.03922071
FBRSL1	12:132493112	G	A	rs12811407	A	0.04879016
RNF6	13:26202862	A	G	rs34584161	A	0.04879016
HMGB1	13:30468315	G	T	rs11842871	G	0.03922071
DLEU1	13:50521959	A	T	rs963740	A	0.03922071
PCDH17	13:57792500	C	T	rs9537803	C	0.03922071
PCDH17	13:58391301	C	T	rs9569864	C	0.04879016
SRGAP2D	13:58503272	A	T	rs9563615	A	0.04879016
SRGAP2D	13:58610100	A	G	rs76251711	G	0.14842001
SPRY2	13:80143021	G	A	rs1359790	G	0.0861777

IRS2	13:109779279	G	A	rs4771648	G	0.03922071
AKAP6	14:32833676	G	T	rs17522122	T	0.03922071
CLEC14A	14:38379215	G	T	rs8017808	G	0.03922071
NRXN3	14:79465698	G	C	rs17836088	C	0.05826891
SMEK1	14:91497378	A	G	rs8010382	G	0.03922071
MARK3	14:103427734	G	T	rs62007683	G	0.03922071
RASGRP1	15:38541832	T	C	rs8032939	C	0.05826891
RASGRP1	15:38580914	T	C	rs34715063	C	0.09531018
LTK	15:41517007	G	A	rs11070332	A	0.04879016
ONECUT1	15:52799356	C	T	rs2456530	T	0.05826891
WDR72	15:53455031	C	G	rs528350911	G	0.2390169
TCF12	15:57164604	A	G	rs117483894	G	0.09531018
C2CD4A/B	15:62102065	G	C	rs8037894	G	0.04879016
USP3	15:63579093	C	T	rs7178762	C	0.03922071
MAP2K5	15:67788548	A	T	rs4776970	A	0.03922071
HMG20A	15:77525786	C	A	rs1005752	A	0.07696104
ITFG3	16:245796	T	C	rs6600191	T	0.05826891
ATP2A1	16:28903896	G	A	rs8046545	G	0.03922071
FAM57B	16:30034468	C	G	rs11642430	G	0.03922071
FAM57B	16:30408063	G	C	rs199795270	C	0.22314355
FTO	16:53468034	G	A	rs4281707	G	0.03922071
FTO	16:53724808	G	A	rs78020297	A	0.0861777
FTO	16:53767042	T	C	rs1421085	C	0.12221763
NFAT5	16:69617963	C	T	rs862320	C	0.03922071
BCAR1	16:75200974	C	A	rs72802342	C	0.15700375
BCAR1	16:75482636	G	C	rs3115960	G	0.0295588
SPG7	16:89497647	T	A	rs12920022	A	0.04879016
ATP1B2	17:7646363	C	T	rs1641523	C	0.04879016
GLP2R	17:9881870	G	C	rs7222481	C	0.03922071
RAI1	17:17758488	A	G	rs4925109	A	0.04879016
MLX	17:42579393	G	C	rs34855406	C	0.04879016
COMMD9	18:38698745	T	C	rs62080313	C	0.05826891
TCF4	18:55383415	A	C	rs72926932	C	0.0861777
WDR7	18:57008153	G	C	rs17684074	G	0.03922071
GRP	18:59208996	G	A	rs9957145	G	0.04879016
MC4R	18:60181136	A	T	rs523288	T	0.04879016
MC4R	18:60389333	C	A	rs74452128	C	0.13976194
INSR	19:7240837	T	C	rs75253922	C	0.04879016
FARSA	19:12927601	G	A	rs3111316	A	0.04879016
TM6SF2	19:19277691	A	T	rs8107974	T	0.09531018
PEPD	19:33399932	C	G	rs10406327	C	0.03922071
TOMM40/APOE	19:44908684	T	C	rs429358	T	0.07696104
GIPR	19:45675403	T	C	rs2238689	C	0.03922071
ZC3H4	19:47065746	G	A	rs3810291	A	0.04879016
NKX2.2	20:21486157	T	C	rs13041756	C	0.05826891
RALY	20:34008898	G	A	rs2268078	A	0.03922071

HNF4A	20:44373081	C	T	rs4810426	T	0.0861777
HNF4A	20:44394715	G	A	rs191830490	G	0.21511138
HNF4A	20:44413724	C	T	rs1800961	T	0.16551444
HNF4A	20:44605008	A	G	rs11696357	A	0.05826891
CEBPB	20:50215598	C	T	rs11699802	C	0.03922071
TSHZ2	20:52607055	A	T	rs34454109	A	0.03922071
GNAS	20:58819573	C	G	rs6070625	G	0.04879016
ZBTB46	20:63819311	T	C	rs6011155	T	0.03922071
TCEA2	20:64061822	G	A	rs59944054	A	0.05826891
MTMR3/ASCC2	22:30213565	A	G	rs6518681	G	0.0861777
YWHAH	22:31952854	C	T	rs117001013	C	0.06765865
EP300	22:41093916	G	A	rs5758223	A	0.03922071
PNPLA3	22:43928850	C	T	rs738408	T	0.04879016
PIM3	22:49963202	C	T	rs1801645	C	0.03922071

ESM Table 8 *SNPs used in the generation of both C-peptide GRS (GRS-C1 and GRS-C2). Position of the SNP is based on build GRCh38 on the positive strand. The effect alleles and corresponding weights were used in the calculations.*

**Gene**	**Previous phenotype**	**POS (GRCh38)**	**REF**	**ALT**	**RsID**	**Effect allele**	**Weight**	**Included in GRS-C2**
INS	T1D	11:2160994	A	T	rs689	T	-0.108	Yes
LMO7	T1D	13:75729796	T	C	rs9573641	C	0.059	Yes
IKZF4	T1D	12:56018703	T	G	rs1701704	G	0.060	Yes
CCR7	T1D	17:40608272	T	A	rs112401631	A	0.280	No
IL10	T1D	1:206676164	G	A	rs77599401	A	0.226	No
RUNX3	T1D	1:24970252	A	C	rs10751776	C	-0.041	No
INPP5B	T1D	1:37881745	A	G	rs12742756	G	0.039	No
GP2	T1D	16:20331769	T	C	rs4238595	C	0.039	No
MAP2K5	T2D	15:67788548	A	T	rs4776970	T	-0.06	Yes
MTND2P8	T2D	9:78744197	C	G	rs11137820	G	-0.054	Yes
BNIPL	T2D	2:58753929	G	T	rs10193538	T	-0.053	Yes
FOCAD	T2D	9:20241071	T	C	rs7867635	C	0.0507	No
HAUS6	T2D	9:19067835	A	G	rs7022807	G	-0.054	No
CDKN2A/B	T2D	9:22134069	G	A	rs10811660	A	-0.062	No
NFAT5	T2D	16:69617963	C	T	rs862320	T	0.044	No
SMARCAD1	T2D	4:94170760	A	G	rs6821438	G	-0.041	No
IGF2BP2	T2D	3:185785668	T	A	rs6780171	A	0.042	No
TSPAN8/LGR5	T2D	12:71129173	G	C	rs1796330	C	-0.039	No
NOTCH2	T2D	1:119984359	C	T	rs1493694	T	0.055	No
HNF4A	T2D	20:44413724	C	T	rs1800961	T	0.091	No
DQB1-DQA2	C-peptide	6:32700662	A	C	rs3135002	C	-0.19	Yes

ESM Table 9 Baseline according to tertiles of GRS-1

Characteristics	Tertile 1 (GRS-1 ≤ 15.4)	Tertile 2 (15.4 < GRS-1 ≤ 16.7)	Tertile 3 (GRS-1 > 16.7)	p-value
**n**	160	159	160	
**Sex** -male %	34	40	38	0.621
**Age-** years	41.5 [28.0, 54.0]	38.0 [28.0, 53.0]	42.0 [29.0, 53.0]	0.825
**BMI-** kg/m2	24.7 [22.9, 27.1]	24.4 [22.1, 27.2]	24.7 [22.7, 27.6]	0.916
**Onset of T1D**, years	21.0 [15.0, 34.5]	19.0 [12.0, 29.0]	20.0 [10.8, 30.0]	0.025
**Duration of T1D**, years	13.0 [5.0, 24.2]	16.0 [5.0, 29.0]	20.0 [8.8, 30.2]	0.009
**GRS-1**	14.4±0.9	16.1±0.4	17.5±0.6	< 0.001
**GRS-2**	12.5±1.6	14.0±1.2	15.5±1.0	< 0.001
**GRS-T2D**	15.0 ±0.6	14.8±0.6	14.9±0.6	0.058
**GRS-C1**	-0.56±0.17	-0.55±0.17	-0.59±0.16	0.045
**GRS-C2**	-0.61±0.12	-0.60±0.12	-0.64±0.10	0.014
**Time in range**, %	68.0 [53.0, 81.0]	69.0 [51.0, 79.0]	64.0 [50.0, 80.5]	0.706
**Time above range**, %	29.0 [15.0, 43.0]	29.0 [16.0, 47.0]	31.0 [17.0, 45.0]	0.638
**Time below range**, %	2.0 [1.0, 4.1]	2.0 [1.0, 4.0]	2.0 [1.0, 4.0]	0.663
**GCV**, %	33.9±7.9	34±7.6	34.2±8.6	0.940
**fasting C-peptide** -nmol/l	0.05 [0.05, 0.11]	0.05 [0.05, 0.10]	0.05 [0.05, 0.06]	0.219
**HbA_{1c}** - %	7.3±3.4	7.2±3.2	7.2±3.1	0.837
**HbA_{1c}**-mmol/mol	55.9±13.9	55.1±11.8	55.7±10.9	0.837
**Fasting Glucose-**mmol/l	8.1 [6.3, 10.6]	8.0 [6.5, 10.6]	8.7 [7.0, 11.2]	0.145
**UCPCR-**nmol/mmol	0.04 [0, 0.51]	0.00 [0.00, 0.30]	0.00 [0.00, 0.40]	0.042
**Fasting Glucagon-**pmol/l	101 [62, 168]	101 [53, 148]	81 [59, 126]	0.080
**Glucagon/glucose ratio**	0.44 [0.28, 0.77]	0.42 [0.23, 0.69]	0.33 [0.22, 0.56]	0.016
**Sensor type**				

1 Dexcom G6- %	76	74	74	0.988
2 Freestyle Libre 2- %	14	1	14	
3 Metronic guardian- %	10	12	12	
**Insulin pump,** Yes, %				0.991
0 No	51	48	51	
1 Manual	31	33	31	
2 Predictive Low-Glucose Suspend	1	1	1	
3 Hybrid closed loop	14	14	14	
4 DIY closed loop	3	4	4	
**Tot. insulin/day** - U	36.0 [27.0, 56.0]	35.0 [25.0, 50.5]	36.7 [28.0, 48.0]	0.363
**Smoking**, Yes, %	15	7	9	0.044
**Alcohol**-U	0.29 [0.00, 0.86]	0.29 [0.00, 0.86]	0.29 [0.00, 0.86]	0.569
**Co-medication** - Any, %	46	38	46	0.288
**Albumin-to-creatinine ratio**-U	0.27 [0.00, 0.82]	0.48 [0.00, 0.90]	0.34 [0.00, 0.80]	0.034
**Retinopathy**- Yes, %	33	30	37	0.455
**CVD**- Yes, %	18	19	16	0.661
**Systolic BP**-mmHg	128.9 ±16.1	129.5±18.1	130.4±16.8	0.742
**EGFR**, CKD-epi	90.0 [88.0, 90.0]	90.0 [90.0, 90.0]	90.0 [87.0, 90.0]	0.413

**The presented values are: mean and standard deviation; median and interquartile range (Q1, Q3); or percentage, as appropriate. Values of UCPCR are defined as undetectable (<0.01 nmol/mmol) or detectable (any value equal to or over 0.01 nmol/mmol).*

ESM Table 10 *Overview of all GRS for only participants with determined European genetic ancestry. Associations between genetic risk scores (GRS) and c-peptide to creatinine ratio (UCPCR) in nmol/mmol, time in range (TIR) in %, time above range in % (TAR), time below range in % (TBR), Glucose coefficient of variance (GCV) in % and HbA_{1c} in mmol/mol. UCPCR was categorized binary in detectable/not detectable, TIR in above and below 70%, TAR in above and below 25%, TBR in above and below 4%. HbA_{1c} and GCV were modelled as continuous outcomes. This table depicts logistic or linear regression models where the odds ratio (for logistic regression) or beta (for linear regression) is expressed per score point of the GRS. Standard statistical notation were used (*p<0.05, **p<0.01, ***p<0.001).*

	**UCPCR (detectable)**	**TIR (≥70%)**	**TBR (<4%)**	**TAR (<25%)**	**GCV**	**HbA_{1c}**
	**OR (95% CI)**	**OR (95% CI)**	**OR (95% CI)**	**OR (95% CI)**	**Beta (95% CI)**	**Beta (95% CI)**
**Model 1 - unadjusted**						
GRS-1	0.80 (0.70, 0.91) ***	0.90 (0.79, 1.03)	0.99 (0.86, 1.14)	0.92 (0.81, 1.05)	0.21 (-0.33, 0.75)	0.09 (-0.67, 0.86)
GRS-2	0.85 (0.77, 0.95) **	0.91 (0.82, 1.01)	0.96 (0.85, 1.07)	0.92 (0.83, 1.03)	0.44 (-0.00, 0.89)	-0.05 (-0.67, 0.58)
GRS-T2D	1.25 (0.92, 1.71)	0.98 (0.72, 1.34)	1.35 (0.96, 1.89)	1.10 (0.81, 1.50)	-1.41 (-2.69, -0.13) *	-0.48 (-2.32, 1.36)
GRS-C1	2.95 (0.98, 8.91)	0.92 (0.30, 2.75)	1.16 (0.35, 3.87)	0.54 (0.18, 1.65)	-0.88 (-5.44, 3.68)	0.29 (-6.28, 6.86)
GRS-C2	5.38 (1.08, 26.73) *	2.12 (0.43, 10.46)	0.58 (0.10, 3.26)	0.61 (0.12, 3.05)	-2.17 (-8.77, 4.43)	-3.28 (-12.80, 6.24)
**Model 2 - adjusted for sex**						
GRS-1	0.80 (0.70, 0.91) ***	0.90 (0.80, 1.03)	0.99 (0.86, 1.14)	0.92 (0.81, 1.05)	0.20 (-0.35, 0.74)	0.10 (-0.66, 0.87)
GRS-2	0.85 (0.77, 0.95) **	0.91 (0.82, 1.01)	0.95 (0.85, 1.07)	0.92 (0.83, 1.03)	0.44 (-0.00, 0.88)	-0.05 (-0.68, 0.57)
GRS-T2D	1.25 (0.92, 1.71)	0.98 (0.72, 1.34)	1.34 (0.95, 1.89)	1.10 (0.81, 1.50)	-1.38 (-2.66, -0.10) *	-0.51 (-2.35, 1.33)
GRS-C1	2.94 (0.98, 8.89)	0.91 (0.30, 2.74)	1.14 (0.34, 3.81)	0.54 (0.18, 1.65)	-0.82 (-5.37, 3.74)	0.17 (-6.40, 6.74)
GRS-C2	5.40 (1.08, 26.84) *	2.13 (0.43, 10.54)	0.59 (0.10, 3.35)	0.61 (0.12, 3.05)	-2.25 (-8.84, 4.33)	-3.14 (-12.65, 6.37)

**Model 3 - adjusted for sex and age at onset**						
GRS-1	0.83 (0.72, 0.97) *	0.92 (0.81, 1.05)	1.01 (0.87, 1.16)	0.94 (0.83, 1.08)	-0.03 (-0.56, 0.49)	0.06 (-0.71, 0.83)
GRS-2	0.92 (0.81, 1.03)	0.93 (0.83, 1.03)	0.97 (0.86, 1.09)	0.95 (0.85, 1.05)	0.20 (-0.24, 0.63)	-0.10 (-0.73, 0.53)
GRS-T2D	1.22 (0.86, 1.72)	0.96 (0.70, 1.31)	1.33 (0.94, 1.87)	1.08 (0.79, 1.47)	-1.23 (-2.46, 0.00)	-0.47 (-2.31, 1.37)
GRS-C1	3.41 (0.99, 11.82)	0.87 (0.29, 2.64)	1.10 (0.33, 3.70)	0.51 (0.16, 1.57)	-0.58 (-4.96, 3.79)	0.27 (-6.30, 6.84)
GRS-C2	9.46 (1.56, 57.57) *	2.18 (0.44, 10.90)	0.59 (0.10, 3.36)	0.61 (0.12, 3.11)	-2.12 (-8.45, 4.21)	-3.15 (-12.66, 6.37)
**Model 4 - adjusted for sex, age at onset and disease duration**						
GRS-1	0.87 (0.74, 1.03)	0.93 (0.81, 1.06)	1.01 (0.88, 1.17)	0.95 (0.83, 1.09)	-0.07 (-0.60, 0.46)	-0.01 (-0.78, 0.77)
GRS-2	0.95 (0.83, 1.08)	0.93 (0.84, 1.04)	0.97 (0.86, 1.10)	0.95 (0.85, 1.06)	0.17 (-0.26, 0.61)	-0.14 (-0.78, 0.49)
GRS-T2D	1.17 (0.80, 1.70)	0.95 (0.70, 1.30)	1.32 (0.94, 1.87)	1.07 (0.78, 1.46)	-1.19 (-2.41, 0.04)	-0.40 (-2.24, 1.43)
GRS-C1	1.93 (0.48, 7.87)	0.80 (0.26, 2.46)	1.05 (0.31, 3.56)	0.44 (0.14, 1.40)	-0.11 (-4.52, 4.29)	0.95 (-5.65, 7.54)
GRS-C2	3.51 (0.47, 26.38)	1.94 (0.38, 9.83)	0.53 (0.09, 3.12)	0.49 (0.09, 2.57)	-1.33 (-7.73, 5.07)	-2.05 (-11.63, 7.53)

ESM Table 11 *Overview of all GRS for only participants with over 7 years of disease duration. Associations between genetic risk scores (GRS) and c-peptide to creatinine ratio (UCPCR) in nmol/mmol, time in range in % (TIR), time above range in % (TAR), time below range in % (TBR), Glucose coefficient of variance (GCV) in % and* HbA_{1c} *in mmol/mol. UCPCR was categorized binary in detectable/not detectable, TIR in above and below 70%, TAR in above and below 25%, TBR in above and below 4%.* HbA_{1c} *and GCV were modelled as continuous outcomes. This table depicts logistic or linear regression models where the odds ratio (for logistic regression) or beta (for linear regression) is expressed per score point of the GRS. Standard statistical notation were used (*p<0.05, **p<0.01, ***p<0.001).*

	**UCPCR (detectable)**	**TIR (≥70%)**	**TBR (<4%)**	**TAR (<25%)**	**GCV**	**HbA_{1c}**
	**OR (95% CI)**	**OR (95% CI)**	**OR (95% CI)**	**OR (95% CI)**	**Beta (95% CI)**	**Beta (95% CI)**
**Model 1- unadjusted**						
GRS-1	0.83 (0.71, 0.97) *	0.96 (0.82, 1.12)	1.04 (0.88, 1.22)	0.96 (0.82, 1.13)	0.05 (-0.53, 0.62)	0.11 (-0.77, 0.99)
GRS-2	0.90 (0.79, 1.02)	0.95 (0.84, 1.08)	1.01 (0.89, 1.15)	0.95 (0.83, 1.07)	0.30 (-0.17, 0.77)	0.15 (-0.56, 0.86)
GRS-T2D	1.16 (0.80, 1.68)	0.97 (0.67, 1.39)	1.47 (1.00, 2.16)	1.08 (0.74, 1.56)	-1.70 (-3.02, -0.37) *	0.11 (-1.95, 2.17)
GRS-C1	1.79 (0.47, 6.86)	1.18 (0.32, 4.37)	0.77 (0.20, 2.98)	0.48 (0.12, 1.86)	0.52 (-4.32, 5.35)	-0.13 (-7.60, 7.34)
GRS-C2	4.36 (0.59, 32.09)	4.04 (0.57, 28.61)	0.34 (0.05, 2.56)	0.65 (0.09, 4.85)	0.01 (-7.19, 7.21)	-4.76 (-15.88, 6.36)
**Model 2 - adjusted for sex**						
GRS-1	0.83 (0.71, 0.97) *	0.96 (0.82, 1.12)	1.04 (0.88, 1.22)	0.96 (0.82, 1.13)	0.03 (-0.54, 0.61)	0.11 (-0.77, 0.99)
GRS-2	0.90 (0.79, 1.02)	0.95 (0.84, 1.08)	1.01 (0.89, 1.15)	0.95 (0.83, 1.07)	0.28 (-0.19, 0.75)	0.15 (-0.56, 0.85)
GRS-T2D	1.16 (0.80, 1.68)	0.97 (0.67, 1.39)	1.49 (1.01, 2.20) *	1.08 (0.74, 1.56)	-1.72 (-3.04, -0.41) *	0.13 (-1.92, 2.18)
GRS-C1	1.78 (0.47, 6.81)	1.17 (0.32, 4.35)	0.74 (0.19, 2.93)	0.48 (0.12, 1.87)	0.56 (-4.24, 5.35)	-0.22 (-7.67, 7.22)
GRS-C2	4.42 (0.60, 32.68)	4.05 (0.57, 28.70)	0.34 (0.04, 2.57)	0.65 (0.09, 4.85)	0.04 (-7.10, 7.18)	-4.69 (-15.78, 6.40)

**Model 3 - adjusted for sex and age at onset**						
GRS-1	0.84 (0.70, 0.99) *	0.96 (0.82, 1.12)	1.04 (0.89, 1.23)	0.96 (0.82, 1.13)	-0.03 (-0.60, 0.55)	0.13 (-0.75, 1.01)
GRS-2	0.94 (0.82, 1.08)	0.95 (0.84, 1.08)	1.02 (0.89, 1.16)	0.94 (0.83, 1.07)	0.21 (-0.26, 0.68)	0.19 (-0.53, 0.90)
GRS-T2D	1.17 (0.78, 1.74)	0.97 (0.67, 1.39)	1.49 (1.01, 2.20) *	1.08 (0.74, 1.56)	-1.74 (-3.05, -0.43) **	0.12 (-1.94, 2.17)
GRS-C1	1.90 (0.44, 8.16)	1.17 (0.32, 4.35)	0.74 (0.19, 2.90)	0.48 (0.12, 1.87)	0.47 (-4.30, 5.25)	-0.26 (-7.71, 7.19)
GRS-C2	4.58 (0.53, 39.42)	4.06 (0.57, 28.80)	0.33 (0.04, 2.51)	0.65 (0.09, 4.86)	0.19 (-6.92, 7.30)	-4.79 (-15.89, 6.31)
**Model 4 - adjusted for sex, age at onset and disease duration**						
GRS-1	0.85 (0.71, 1.03)	0.95 (0.81, 1.11)	1.04 (0.88, 1.22)	0.95 (0.81, 1.12)	0.00 (-0.57, 0.58)	0.13 (-0.76, 1.01)
GRS-2	0.95 (0.83, 1.10)	0.95 (0.83, 1.07)	1.01 (0.89, 1.16)	0.94 (0.83, 1.07)	0.23 (-0.25, 0.70)	0.18 (-0.53, 0.90)
GRS-T2D	1.10 (0.73, 1.66)	0.98 (0.68, 1.41)	1.50 (1.01, 2.21) *	1.09 (0.75, 1.57)	-1.78 (-3.09, -0.47) **	0.13 (-1.93, 2.19)
GRS-C1	1.29 (0.28, 5.92)	1.30 (0.35, 4.89)	0.77 (0.19, 3.07)	0.51 (0.13, 2.00)	0.12 (-4.69, 4.94)	-0.18 (-7.70, 7.33)
GRS-C2	2.23 (0.24, 21.04)	5.08 (0.70, 37.03)	0.35 (0.05, 2.74)	0.73 (0.09, 5.56)	-0.48 (-7.69, 6.73)	-4.74 (-15.96, 6.48)
